# LC3C, Bound Selectively by a Noncanonical LIR Motif in NDP52, Is Required for Antibacterial Autophagy

**DOI:** 10.1016/j.molcel.2012.08.024

**Published:** 2012-11-09

**Authors:** Natalia von Muhlinen, Masato Akutsu, Benjamin J. Ravenhill, Ágnes Foeglein, Stuart Bloor, Trevor J. Rutherford, Stefan M.V. Freund, David Komander, Felix Randow

**Affiliations:** 1 MRC Laboratory of Molecular Biology, Division of Protein and Nucleic Acid Chemistry, Hills Road, Cambridge CB2 0QH, UK

## Abstract

Autophagy protects cellular homeostasis by capturing cytosolic components and invading pathogens for lysosomal degradation. Autophagy receptors target cargo to autophagy by binding ATG8 on autophagosomal membranes. The expansion of the ATG8 family in higher eukaryotes suggests that specific interactions with autophagy receptors facilitate differential cargo handling. However, selective interactors of ATG8 orthologs are unknown. Here we show that the selectivity of the autophagy receptor NDP52 for LC3C is crucial for innate immunity since cells lacking either protein cannot protect their cytoplasm against *Salmonella*. LC3C is required for antibacterial autophagy because in its absence the remaining ATG8 orthologs do not support efficient antibacterial autophagy. Structural analysis revealed that the selectivity of NDP52 for LC3C is conferred by a noncanonical LIR, in which lack of an aromatic residue is balanced by LC3C-specific interactions. Our report illustrates that specificity in the interaction between autophagy receptors and autophagy machinery is of functional importance to execute selective autophagy.

## Graphical abstract

**Graphical Abstract:**

**Figure:**
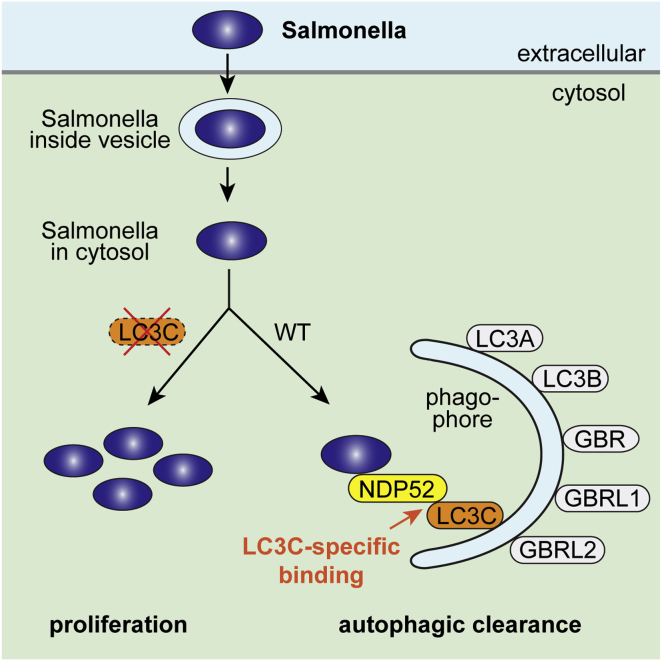

**Highlights:**

► The autophagy receptor NDP52 binds selectively the ATG8 family member LC3C ► A noncanonical LIR motif in NDP52 provides specificity for LC3C ► LC3C and NDP52 are required for autophagy and restriction of *S.* Typhimurium ► LC3C required to recruit all ATG8 orthologs into antibacterial autophagosomes

## Introduction

Autophagy is a lysosome-dependent degradation pathway for cytosolic components that regulates physiological and pathophysiological processes, for example the response to starvation, the destruction of protein aggregates and the defense against pathogens (Mizushima et al., 2008; Levine et al., 2011; Mizushima et al., 2011). Macroautophagy, hereafter referred to as autophagy, relies on de novo-generated autophagosomes, which are double-membrane organelles that engulf cytosol for delivery to lysosomes (in mammals) or the vacuole (in yeast).

In yeast more than thirty AuTophaGy (ATG) genes are known, most of which are essential for autophagy and have orthologs in higher eukaryotes (Mizushima et al., 2011). Autophagy requires two ubiquitin-like conjugation systems; one involving the conjugation of ATG12 onto ATG5, the other the conjugation of ATG8 onto phosphatidylethanolamine in the autophagosomal membrane (Mizushima et al., 1998; Ichimura et al., 2000; Geng and Klionsky, 2008). ATG8 remains associated with mature autophagosomes and is therefore used as a bona fide marker for this organelle (Klionsky et al., 2008). ATG8 is essential for autophagy in yeast (Lang et al., 1998; Kirisako et al., 1999), although its exact function remains to be determined. ATG8 is required for phagophore expansion to which the membrane tethering and hemifusion activity of ATG8 may contribute (Nakatogawa et al., 2007; Weidberg et al., 2011a). Additional SNARE proteins are required for the fusion of membranes with low PE content (Moreau et al., 2011; Nair et al., 2011).

While *S. cerevisiae* encodes only a single ATG8 gene, the genomes of plants, metazoans and certain protists harbor multiple, in some cases up to 25 ATG8 orthologs (Williams et al., 2009; Shpilka et al., 2011). The human genome contains six functional ATG8 orthologs and several pseudogenes. LC3A, LC3B, and LC3C form the LC3 subfamily, while GABARAP, GABARAPL1 (GEC1), and GABARAPL2 (GATE16) form the GABARAP subfamily. All mammalian orthologs associate with autophagosomes (Kabeya et al., 2004) but little is known about the specific function of individual family members except that during starvation-induced autophagy the LC3 family is required for the elongation of autophagosomal membranes while the GABARAP family functions further downstream in autophagosome biogenesis (Weidberg et al., 2010).

Starvation-induced autophagy is a nonselective process (Weidberg et al., 2011b). In contrast, the engulfment of aggregated proteins, damaged mitochondria, cytosol-invading pathogens and other specific cytosolic components is selectively mediated by autophagy receptors (Johansen and Lamark, 2011). Autophagy receptors rely on ‘eat-me’ signals to specify cytosolic components as autophagy cargo, such as polyubiquitin or Galectin-8 bound to glycans on damaged vesicles (Kirkin et al., 2009b; Thurston et al., 2012). By binding ATG8 family members, autophagy receptors physically link their cargo and the phagophore membrane. The binding of autophagy receptors to ATG8 depends on the formation of an intermolecular β sheet, to which the autophagy receptor contributes a single strand, the so-called ATG8/LC3-interacting region (LIR) (Noda et al., 2008). The consensus LIR motif Trp/Phe-Xaa-Xaa-Leu/Ile/Val is preceded by acidic residues (Johansen and Lamark, 2011). Phosphorylation can enhance the affinity of the interaction (Wild et al., 2011).

The human genome encodes at least four ubiquitin-binding autophagy receptors, i.e., p62 (SQSTM1) (Bjørkøy et al., 2005), NBR1 (Kirkin et al., 2009a), NDP52 (Thurston et al., 2009), and Optineurin (Wild et al., 2011). A recent proteomics study identified 67 candidate proteins that interact with the human ATG8 orthologs (Behrends et al., 2010). Although the well-characterized autophagy receptors p62, NBR1, and Optineurin bind nonspecifically to multiple LC3/GABARAP proteins (Pankiv et al., 2007; Kirkin et al., 2009a; Wild et al., 2011), the expansion of the ATG8 family and the occurrence of many LC3/GABARAP-binding proteins in higher eukaryotes suggests that a network of selective interactions could provide functionally important specificity to allow the differential handling of autophagic cargo.

Selective autophagy is essential for the defense of the mammalian cytosol against bacterial invasion (Deretic and Levine, 2009; Fujita and Yoshimori, 2011; Randow, 2011; Randow and Münz, 2012). Consequently, professional cytosol-dwelling bacteria, such as *Shigella* (*S. flexneri*), avoid autophagy, while *Salmonella enterica* serovar Typhimurium (*S.* Typhimurium), which is less adapted to life in the cytosol, is restricted by autophagy in its ability to proliferate (Ogawa et al., 2005; Birmingham et al., 2006). Autophagy of *S.* Typhimurium occurs because membranes damaged by bacteria entering the cytosol attract Galectin-8 (Thurston et al., 2012) and because the pathogen, once exposed to the cytosol, becomes decorated with polyubiquitin (Perrin et al., 2004). Galectin-8 is specifically sensed by NDP52, while the ubiquitin coat is detected by three nonredundant autophagy receptors, i.e., NDP52, p62, and Optineurin (Thurston et al., 2009, 2012; Zheng et al., 2009; Wild et al., 2011).

Here we show that NDP52 binds selectively to LC3C and that the interaction is important for innate immunity since cells lacking either protein or expressing alleles unable to bind their respective ligand fail to protect their cytoplasm against invasion by *S.* Typhimurium. NDP52 and LC3C are required for antibacterial autophagy because in their absence the remaining ATG8 orthologs do not support efficient engulfment of bacteria into autophagosomes. The selectivity of NDP52 for LC3C is conferred by a noncanonical LIR motif, termed CLIR, which comprises the tripeptide Leu-Val-Val. The lack of an aromatic residue in the CLIR is balanced by several specific interactions in the periphery of the CLIR-docking site in LC3C. Our report indicates that specificity in the interaction between autophagy receptors and the autophagy machinery is of functional importance to execute autophagy.

## Results

### NDP52 Binds Selectively to LC3C

**Figure 1:**
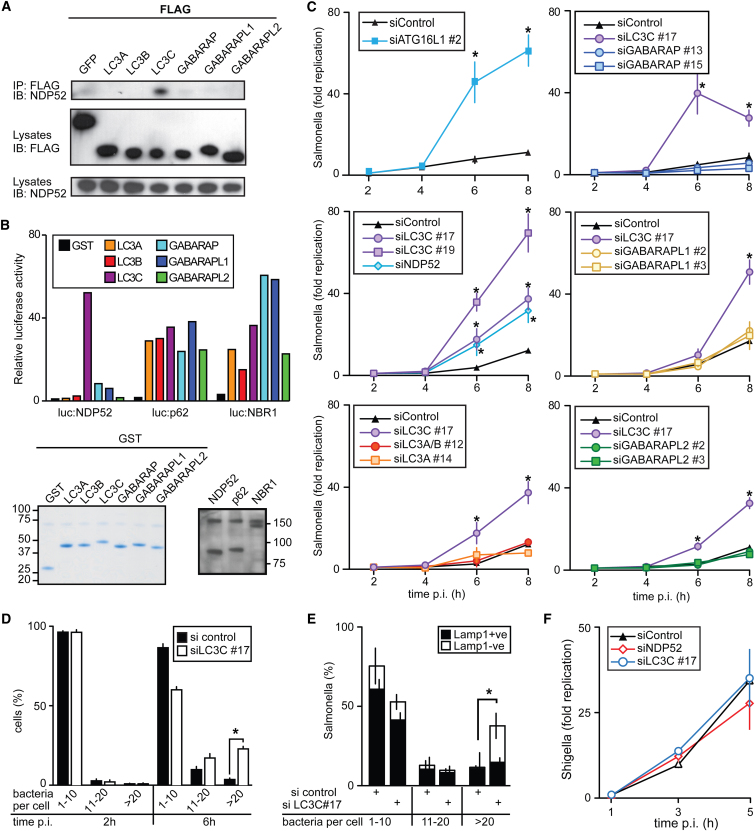
Selective Binding of LC3C by NDP52 and Requirement for LC3C in Antibacterial Autophagy (A) Lysates of 293ET cells expressing the indicated FLAG-tagged proteins were immunoprecipitated with anti-FLAG beads. Precipitates and lysates were probed with the indicated antibodies. (B) LUMIER binding assay. Normalized ratio between luciferase activity bound to beads and present in lysates. Upper: Lysates of 293ET cells expressing NDP52, p62, or NBR1 each fused to luciferase, incubated with purified GST-tagged ATG8 orthologs bound to beads. Lower: GST-fusion proteins stained with Coomassie and lysates of 293ET cells probed with anti-luciferase antibody. (C) Kinetics of *S*. Typhimurium replication in HeLa cells transfected with the indicated siRNAs. Bacteria counted on the basis of their ability to form colonies on agar plates. SiRNAs are further characterized in Figure S1. (D and E) Hyperproliferation of *S*. Typhimurium in a LAMP1-negative compartment despite normal infectivity in cells depleted of LC3C. HeLa cells transfected with the indicated siRNAs and infected with S. Typhimurium. (D) Percentage of cells containing the indicated number of bacteria at the indicated time points. (E) Percentage of LAMP1-positive and -negative bacteria, identified by antibody staining at 6 hr post infection (p.i.). Cells binned according to the number of bacteria they contained. (F) Kinetics of *S. flexneri* replication in HeLa cells transfected with the indicated siRNAs. Bacteria counted on the basis of their ability to form colonies on agar plates. (All panels) Mean and standard deviation (SD) of triplicate HeLa cultures and duplicate colony counts. Mean and SD of triplicate cultures. ^∗^p < 0.05, Student's t test.

Ubiquitin and ubiquitin-like proteins share a common fold despite their low sequence conservation. Pairwise sequence identities between the human ATG8 orthologs range from 31% to 87%, indicating less conservation among some ATG8 orthologs than between ubiquitin and NEDD8 (58%), which suggests that certain ATG8 orthologs may have acquired specific functions (Table 1 and Figure S1A). We therefore investigated whether autophagy receptors would bind selectively to human ATG8 orthologs. Using immunoprecipitation and LUMIER analysis (Barrios-Rodiles et al., 2005), we found that NDP52 bound preferentially to LC3C (Figures 1A and 1B). In contrast, p62 and NBR1 interacted nonselectively with all human ATG8 orthologs, as reported earlier (Pankiv et al., 2007; Kirkin et al., 2009a). Because NDP52 expressed in *E. coli* also bound preferentially to purified GST-LC3C (Figure S1B), NDP52 possesses an inherent specificity for LC3C independent from the potential contribution of other eukaryotic proteins or posttranslational modifications.

### LC3C Is Essential for Efficient Restriction of *S.* Typhimurium

NDP52 protects the cytosol against invasion by *S.* Typhimurium by targeting galectin-8- and ubiquitin-decorated bacteria for autophagy (Thurston et al., 2009; 2012). To test whether the specific NDP52 ligand LC3C is also required for the antibacterial defense of cells, we depleted cells of NDP52 or LC3C. We confirmed that cells lacking ATG16, a core autophagy gene, or NDP52 fail to restrict proliferation of *S.* Typhimurium (Figure 1C). Importantly, cells depleted of LC3C also did not restrict the proliferation of *S.* Typhimurium, while individual depletion of other ATG8 orthologs had no effect (Figures 1C and S1C–S1F). Even the simultaneous depletion of LC3A and LC3B with siLC3A/B#12, a fortuitously crossreactive siRNA, did not result in hyperproliferation. Microscopic analysis confirmed that the greater bacterial burden of cells treated with siRNAs against NDP52 or LC3C was due to bacterial hyperproliferation rather than differences in the uptake of bacteria (Figure 1D). Hyperproliferation in LC3C-depleted cells occurred in a LAMP1-negative compartment, consistent with proliferation of bacteria in the cytosol due to a defect in autophagosomal uptake (Figure 1E). As an additional test for the specificity of the LC3C phenotype we infected siRNA-treated cells with *S. flexneri*, a professional cytosol-dwelling pathogen that is not restricted by NDP52 (Thurston et al., 2009) (Figure 1F). As expected, depletion of LC3C or NDP52 did not impact on the proliferation of *S. flexneri*. We therefore conclude that both NDP52 and its specific interaction partner LC3C are required to defend the cytosol against invasion by *S.* Typhimurium.

### All Human ATG8 Orthologs Colocalize with *S.* Typhimurium

**Figure 2:**
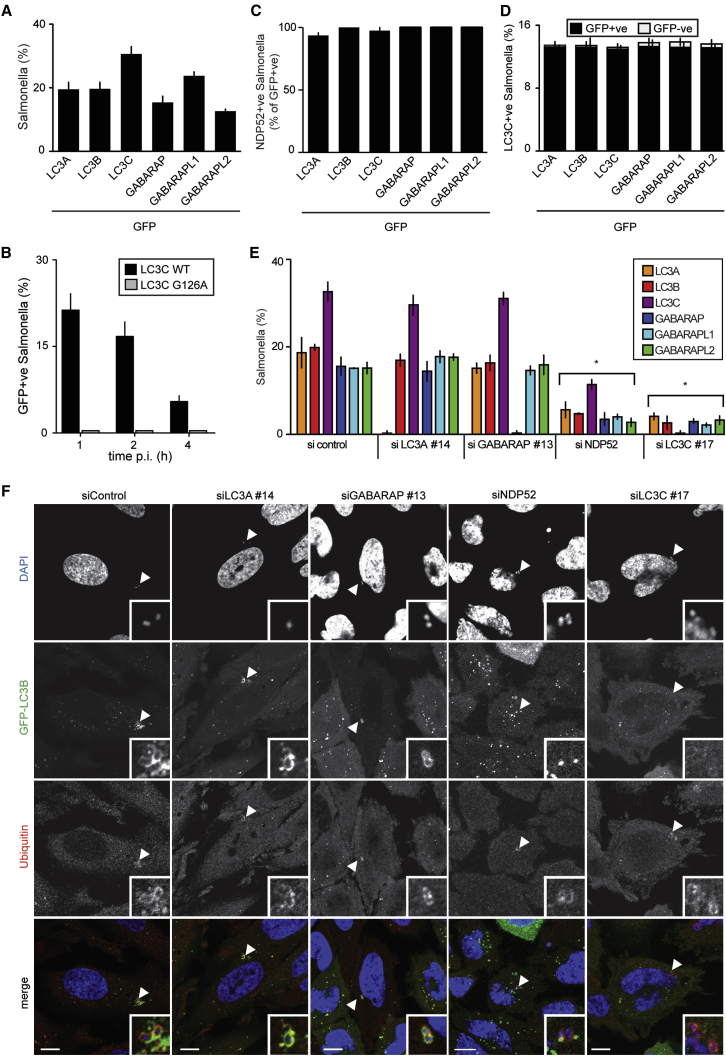
LC3C Is Required to Recruit Other ATG8 Orthologs to *S*. Typhimurium (A–D) Analysis of HeLa cells stably expressing the indicated GFP-tagged ATG8 orthologs and infected with *S*. Typhimurium. Percentage of bacteria coated at 1 hr p.i. with the indicated ATG8 orthologs (A) and the indicated LC3C alleles (B). Percentage of GFP-positive bacteria stained by an antibody for NDP52 at 1 hr p.i. (C) and percentage of mCherry-LC3C positive bacteria (D). (E and F) Analysis of HeLa cells stably expressing the indicated GFP-tagged ATG8 orthologs, treated with the indicated siRNAs, and infected with *S*. Typhimurium. (E) Percentage of bacteria coated by the indicated ATG8 orthologs at 1 hr p.i. (F) Confocal microphotographs of HeLa cells expressing GFP-tagged LC3B stained with DAPI and an antibody against ubiquitin. siRNAs are further characterized in Figure S1. (All panels) Mean and SD of triplicate cultures. ^∗^p < 0.05, Student's t test.

The importance of LC3C in restricting bacterial growth is, to the best of our knowledge, the first demonstration of a nonredundant role for an individual ATG8 ortholog. To investigate the function of NDP52 and LC3C in further detail we tested which ATG8 orthologs are recruited to those *S.* Typhimurium that during host cell invasion have been released into the cytosol. Upon infection of HeLa cells all members of the LC3- and GABARAP-subfamilies colocalized with about 20% of *S.* Typhimurium, a number typical for the cytosol-exposed fraction of intracellular *S.* Typhimurium (Figure 2A) (Thurston et al., 2009). Recruitment of LC3C required its lipidation, as LC3C^G126A^, a lipidation-resistant mutant, was not recruited to *S.* Typhimurium (Figures 2B and S2A). NDP52 as well as LC3C colocalized extensively with all ATG8 orthologs on bacteria (Figures 2C, 2D, S2B, and S3). We conclude that autophagosomes enwrapping *S.* Typhimurium contain multiple ATG8 orthologs and that LC3C, despite its selective interaction with NDP52, does not label a distinct subpopulation of autophagy-targeted *S.* Typhimurium. Rather, LC3C performs a function in antibacterial autophagy that other ATG8 family members, despite their presence on the same autophagy-targeted bacterium, cannot execute.

### LC3C Is Required for the Recruitment of All ATG8 Family Members to *S.* Typhimurium

Selective autophagy relies on cargo-binding autophagy receptors that interact with ATG8 orthologs on the isolation membrane (Johansen and Lamark, 2011). Since cells depleted of NDP52 fail to deliver *S.* Typhimurium into autophagosomes (Thurston et al., 2009), we investigated which ATG8 orthologs depend on NDP52 for their accumulation around cytosol-exposed bacteria. In contrast to control siRNA-treated cells, in cells depleted of NDP52 none of the ATG8 orthologs colocalized efficiently with *S.* Typhimurium (Figures 2E and 2F). Depletion of LC3C caused the same phenotype, i.e., none of the ATG8 orthologs was recruited efficiently to *S.* Typhimurium in the absence of LC3C. In contrast, cells depleted of LC3A or GABARAP had no defect in the recruitment of ATG8 orthologs to *S.* Typhimurium (Figures 2E and 2F). We conclude that NDP52 specifically recruits LC3C to *S.* Typhimurium, and that both proteins are essential for efficient recruitment of the remaining ATG8 orthologs. In line with the crucial role of LC3C in restricting bacterial growth (Figure 1C), we propose that LC3C performs an essential function in antibacterial autophagy downstream of NDP52, namely the formation of functional autophagosomes containing all ATG8 orthologs.

### Identification of the LC3C-Specific LIR in NDP52

**Figure 3:**
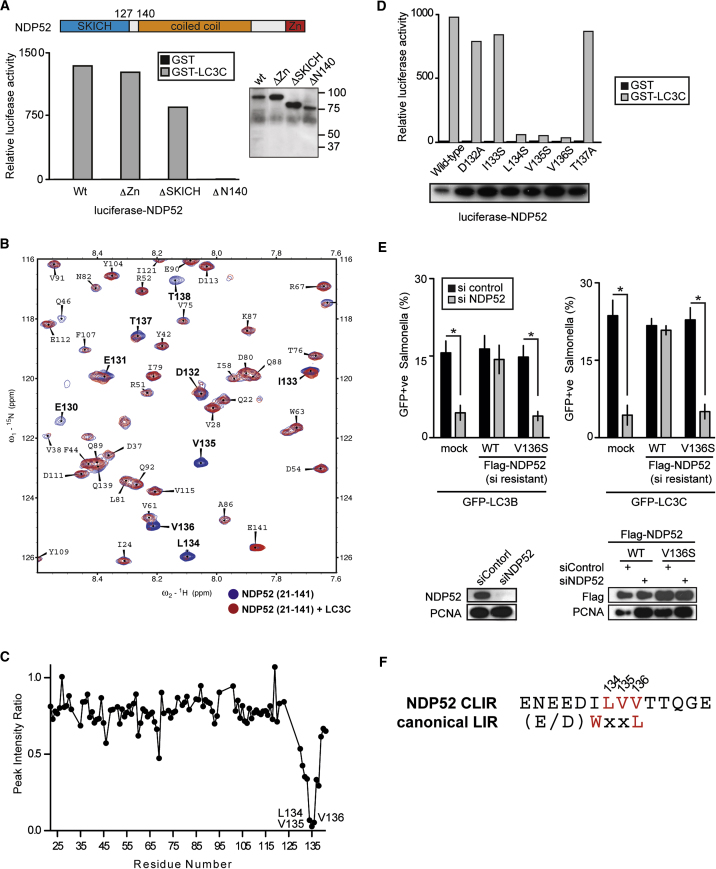
Mapping the NDP52 LC3C Interacting Region (A) Upper: Domain structure of NDP52. Zn, zinc finger ubiquitin-binding domain. Lower: LUMIER binding assay. Normalized ratio between luciferase activity bound to beads and present in lysates. Lysates of 293ET cells expressing the indicated NDP52 alleles fused to luciferase incubated with the indicated purified GST proteins bound to beads. Blot: Lysates of 293ET cells probed with anti-luciferase antibody. (B) Section of fully assigned ^15^N,^1^H-HSQC spectra of ^13^C,^15^N-labeled NDP52 (aa 21–141, 150 μM) domain with (red) and without (blue) 150 μM unlabeled LC3C (aa 1–126) present. Resonances labeled in bold are exchange-broadened upon addition of LC3C (see Figure S4 for full spectra). (C) Indication of exchange broadening in NDP52 (aa 21–141, 150 μM) upon addition of 60 μM LC3C, derived from the peak intensity ratios. See Figure S5 for further titration points obtained over LC3C concentrations ranging from 5 to 150 μM. (D) LUMIER binding assay. Normalized ratio between luciferase activity bound to beads and present in lysates. Lysates of 293ET cells expressing the indicated NDP52 alleles fused to luciferase were incubated with the indicated purified GST proteins bound to beads. Blot: Lysates of 293ET cells probed with anti-luciferase antibody. (E) Percentage of GFP-positive S. Typhimurium at 1 hr p.i. in HeLa cells stably expressing siRNA-resistant NDP52 alleles and treated with the indicated siRNAs. Lower: Lysates of HeLa cells probed with the indicated antibodies. (F) Alignment of the NDP52 CLIR sequence and the canonical LIR consensus motif. Numbers correspond to human NDP52. (All panels) Mean and SD of triplicate cultures. ^∗^p < 0.05, Student's t test.

To better understand the requirement for LC3C in antibacterial autophagy we investigated which region in NDP52 confers LC3C specificity. NDP52 comprises an N-terminal SKICH domain of unknown structure (aa 1–127), a central coiled-coil forming region (aa 140–420) and a C-terminal ubiquitin-binding Zn finger domain (ZnF, aa 421–446) (Figure 3A). Deletion of the ZnF (ΔZn) or the SKICH domain (ΔSKICH) did not disrupt NDP52 binding to LC3C. In contrast, the LC3C interaction was lost when the first 140 aa were deleted (NDP52 ΔN140, aa 141–446) (Figure 3A). The 13 aa stretch connecting SKICH and coiled-coil domain is therefore required for binding of LC3C, suggesting it may contain the LC3C-interacting region (CLIR).

To understand the contribution of the CLIR containing linker region in molecular detail, we reverted to nuclear magnetic resonance (NMR) experiments. In heteronuclear correlation (^15^N,^1^H-HSQC) spectra of the N-terminal region of NDP52 (aa 21–141), 109 of 116 correlations from non-Pro residues were assigned using standard triple resonance experiments (Figures 3B and S4).

Addition of unlabeled LC3C to ^15^N-labeled NDP52 (both at 150 μM) resulted in intensity attenuation of several resonances in the C-terminal region of NDP52, i.e., residues 130–140 (Figure 3B), indicating specific interaction of LC3C with this region, consistent with domain mapping data (Figure 3A). Quantification of signal intensities revealed that three residues, Leu134, Val135, and Val136, were attenuated most severely, suggesting they directly contribute to LC3C binding (Figure 3C). This conclusion was confirmed in titration experiments in which the LC3C concentration was varied from 5 to 150 μM (Figure S5). In contrast, resonances in the SKICH domain were unperturbed upon addition of LC3C, indicating that no secondary interface between the SKICH domain and LC3C exists (Figures 3C and S5).

To identify residues essential for the binding of LC3C in vitro, amino acids 132–137 of NDP52 were mutated individually to Ala or Ser in the context of full-length protein. Mutations of Leu134, Val135, or Val136, but not of Ile133 or other surrounding residues abrogated the interaction of NDP52 with LC3C in a LUMIER assay (Figure 3D).

To test the importance of the NDP52-LC3C interaction in vivo, we complemented cells lacking NDP52 with NDP52 alleles that differ in their ability to bind LC3C. Recruitment of LC3C and LC3B to *S.* Typhimurium was re-established upon complementation with wild-type NDP52 but not with NDP52 V136S, which demonstrates the crucial importance of the NDP52-LC3C interaction for antibacterial autophagy (Figure 3E). These data, in combination with our NMR measurements, define the tripeptide Leu-Val-Val in the linker region between SKICH and coiled-coil domain as the essential CLIR motif for binding to LC3C. This tripeptide is predicted to form a β strand, like the canonical Trp-Xaa-Xaa-Leu LIR motif, but lacks the aromatic residue found in canonical LIR motifs (Figure 3F).

### Structure of the NDP52 SKICH Domain

**Figure 4:**
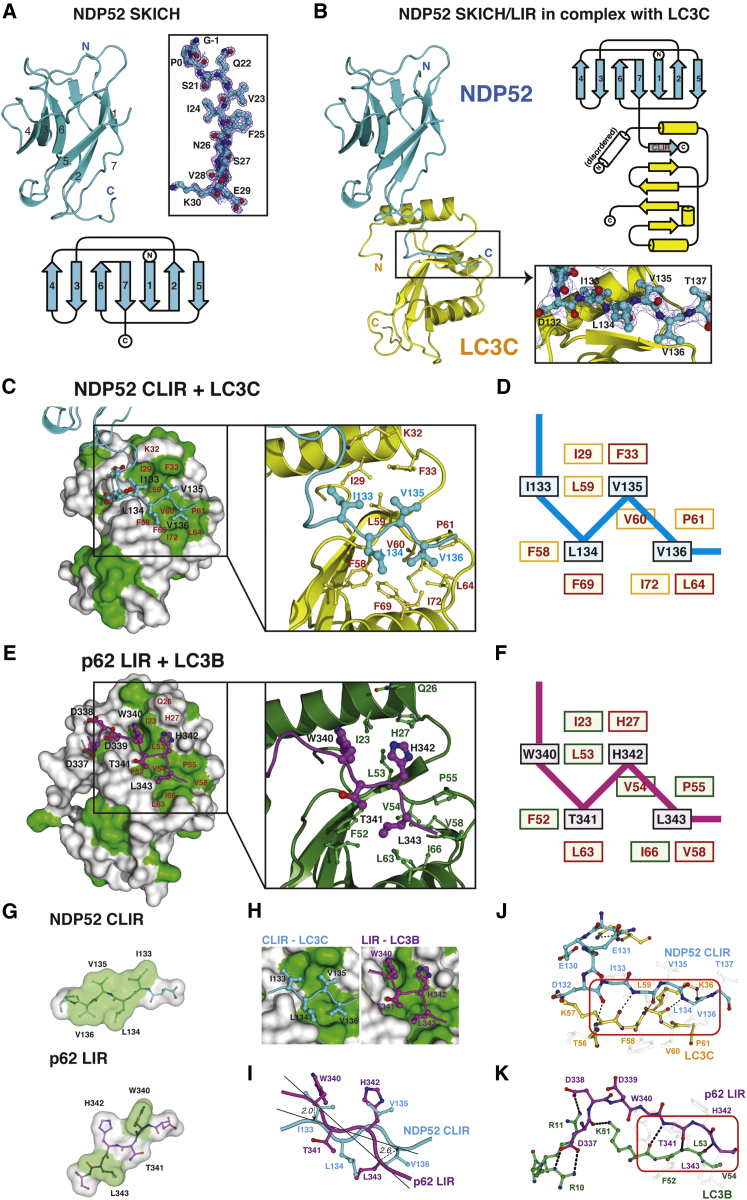
Origin of LC3C Specificity in NDP52 (A) Crystal structure of the NDP52 SKICH domain (aa 21-141) in cartoon representation and as topology diagram. N and C termini are indicated and β strands are numbered. Inset: The first β strand in ball-and-stick representation with a 2|F_o_|-|F_c_| electron density (blue mesh) contoured at 1σ. (B) Structure of NDP52 21–141 (cyan) in complex with LC3C (yellow). NDP52 in the same orientation as in (A). Inset: Residues of the CLIR with electron density as in (A). Right: Topology diagram. (C) Interaction of NDP52 CLIR and LC3C. Hydrophobic residues (Phe, Leu, Val, Ile, Pro) of LC3 in green. Inset: NDP52 (cyan) and LC3C (yellow, red labels). (D) Schematic diagram of interactions between LC3C and the NDP52 CLIR. Residues in red boxes differ between LC3C and LC3B (compare Figure 4F). (E) Interaction of p62 LIR and LC3B (pdb-id 2zjd, (Ichimura et al., 2008)), shown as in (C) with a purple p62 LIR peptide. (F) Schematic diagram of interactions between LC3B and the canonical LIR of p62. Residues in red boxes differ between LC3C and LC3B (compare Figure 4D). (G) NDP52 CLIR and p62 LIR peptides under a semitransparent surface (colored as in C, in an orientation as seen by LC3. (H) Close-up of the CLIR (left) and LIR (right) interactions with LC3 under a surface as in (D) and (F), respectively. (I) CLIR and LIR peptides upon superposition of LC3 molecules in NDP52-LC3C and p62-LC3B complexes. Dotted lines distances in Å. (J–K) Hydrogen bonds of the NDP52 CLIR (J) and the p62 LIR (K). Backbone hydrogen bonds boxed.

Further insight into the specific recognition of LC3C by NDP52 came from crystallographic analysis. We determined the crystal structure of the NDP52 N terminus (aa 21–141) containing the SKICH domain and CLIR in isolation and in complex with LC3C (Figure 4A and 4B and Table 2). Since the SKICH domain fold was unknown, experimental phasing was performed exploiting the anomalous scattering of cadmium ions contained in one of two independent crystal forms. Single anomalous dispersion (SAD) phasing of a 2.7 Å data set was used to build an initial model, which served in molecular replacement to determine the 1.35 Å crystal structure of the NDP52 SKICH domain in a different space group (Table 2). The structure revealed a β sandwich fold comprising seven antiparallel β strands, organized in two sheets of four and three strands (Figure 4A). Some loops connecting the seven β strands of NDP52 fold back to form hydrophobic interactions with the β sheets. The SKICH domain resembles an Ig-fold lacking the eighth β strand, which places the N and C termini onto opposite sides of the β sandwich. The closest structural homolog of the SKICH domain is present in the human C3 complement component, which is comprised of multiple Ig and Ig-like folds (pdb-id 2a74, (Janssen et al., 2005), DALI Z-score 8.3).

### Structure of the NDP52–LC3C-Complex

The NDP52 CLIR was disordered in crystals of apo-NDP52 but became structured in the 2.5 Å complex structure of NDP52 bound to LC3C (Figure 4B). Similar to other LC3-LIR complexes (Noda et al., 2010) and to SUMO-SIM interactions (Hecker et al., 2006), the CLIR forms a short β strand (Figure 4B) that fits into the canonical, hydrophobic LIR binding groove of LC3C (Figures 4C and 4D). As expected from the NMR data and mutational analysis (Figure 3), the interaction is driven by extensive hydrophobic interactions of Leu134, Val135, and Val136.

Several structures of ATG8 orthologs bound to canonical LIR motifs have been analyzed (Noda et al., 2010), among which is a crystal structure of LC3B bound to the p62 LIR peptide (pdb-id 2zjd; Ichimura et al., 2008) (Figures 4E and 4F). This structure is representative for the interaction of ATG8 orthologs with canonical LIR motifs. By comparing the LC3B-LIR^p62^ complex with our LC3C-CLIR^NDP52^ structure, three significant differences became apparent (Figures 4G–4K).

### Origin of LC3C-Specificity in NDP52: A Distinct Footprint of LIR and CLIR

**Figure 5:**
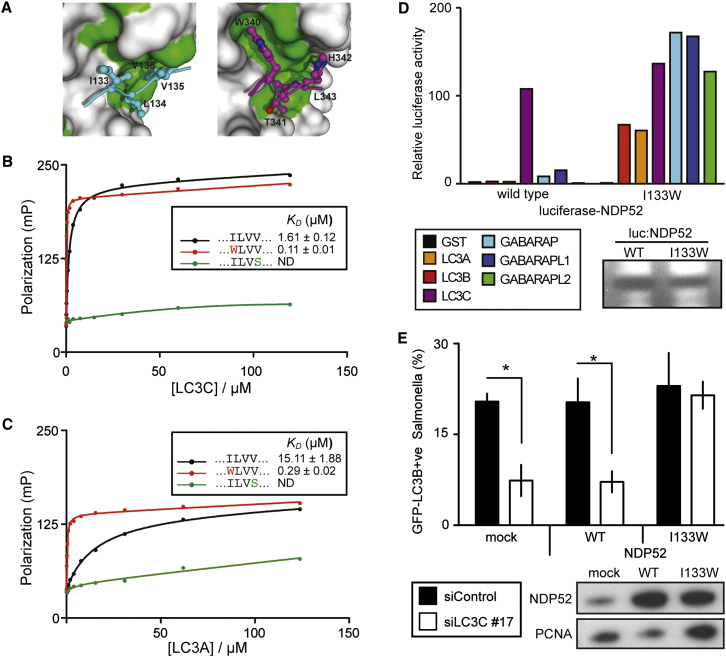
Manipulation of NDP52 CLIR Affinity and Specificity (A) Detail of the CLIR/LC3C (top) and LIR/LC3B (bottom) interactions with the aromatic pocket in a different orientation than in Figure 4H. (B–C) Fluorescence anisotropy assay of FITC-labeled NDP52 CLIR peptides (aa 128–141) against LC3C (B) and LC3A (C). Errors in *K*_*D*_ represent standard deviation. (D) LUMIER binding assay. Normalized ratio between luciferase activity bound to beads and present in lysates. Lysates of 293ET cells expressing the indicated NDP52 alleles fused to luciferase, incubated with purified GST-tagged ATG8 orthologs bound to beads. Blot: Lysates probed with anti-luciferase antibody. (E) Analysis of HeLa cells stably expressing GFP-tagged LC3B, treated with the indicated siRNAs, transduced with the indicated NDP52 alleles, and infected with *S*. Typhimurium for 1 hr. Percentage of bacteria coated by LC3B. Blot: Lysates of cells expressing the indicated siRNA-resistant NDP52 alleles and treated with siLC3C#17 were probed with anti-NDP52 antibody. (All panels) Mean and SD of triplicate cultures. ^∗^p < 0.05, Student's t test.

The most notable difference between the CLIR and LIR motifs is the lack of an aromatic residue in the CLIR peptide. In the LC3B-p62 complex, Trp340 at position 1 of the LIR β strand (sequence Trp-Thr-His-Leu) is buried in a deep, hydrophobic pocket, termed the aromatic, or Trp pocket (Noda et al., 2010) (Figures 4E and 4H) that is conserved in all mammalian ATG8 orthologs, including LC3C. Ile133 of the NDP52 CLIR β strand is at the Trp340-equivalent position but due to its shorter side chain does not occupy the aromatic pocket and is therefore largely solvent exposed (Figures 4H and 5A). Consistently, while mutation of the aromatic residue in LIR motifs abolishes LC3 interactions (Ichimura et al., 2008; Noda et al., 2008), mutation of Ile133 in NDP52 had no effect on binding to LC3C (Figure 3D).

As noted above, and in contrast to canonical LIR β strands where the first and fourth residue mediate binding, the CLIR-LC3C interaction is dependent on three consecutive hydrophobic residues, the Leu-Val-Val motif, which occupy position two, three, and four of the CLIR β strand. Despite pointing to opposite sides of the β strand, these residues form an extensive, flat hydrophobic surface, with Leu134 and Val136 on one, and Val135 on the other side. The hydrophobic CLIR motif is opposed by a complementary hydrophobic surface on LC3C that incorporates the so-called Leu-pocket in Atg8 family members (Noda et al., 2010) (Figures 4C and 4D). The Leu-pocket buries the conserved leucine in position four of canonical LIR β strands. However, the intermittent residues (i.e., Thr341, His342 in p62; Figure 4E) do not contribute significantly to ATG8 binding (Ichimura et al., 2008; Noda et al., 2008). This situation contrasts the CLIR-LC3C interaction, in which three adjacent residues form hydrophobic interactions. Val136 in position four and Leu134 in position two of the CLIR β strand cooperate to occupy a complementary, smooth hydrophobic surface on LC3C in which the Leu-pocket is less pronounced than in other ATG8 family members. The side chain of Leu134 points toward Val136, and while neither residue occupies the Leu position in a canonical LIR (Leu343 in p62), they together interact with residues forming the Leu-pocket in LC3C and additional surrounding residues (Figures 4C, 4E, 4H, and 4I). In addition, Val135 on the opposite face of the β strand exploits another hydrophobic wall of the LC3C binding pocket (Figures 4C, 4D, and 4H), formed by Phe33.

Therefore, as a result of the distinct hydrophobic footprints of CLIR and LIR, their interaction modes are significantly different (Figure 4G). In contrast to canonical LIRs, which bind with a two-pronged mechanism, the CLIR in NDP52 binds a larger, but flat, hydrophobic area.

### Origin of LC3C-Specificity in NDP52: CLIR Rotation Optimizes Hydrogen Bonding

Despite their distinct binding modes, the CLIR and LIR β strands span an identical distance (Figures 4H and 4I). The key difference between the binding modes of the peptides is a rotation of ∼24° around Leu134 of the CLIR (Figure 4I). This rotation of the CLIR β strand moves Ile133 away from the aromatic pocket. However, it also improves shape complementarity with the hydrophobic CLIR pocket, and enables additional interstrand hydrogen bonds between the CLIR of NDP52 and the β2-strand of LC3C (Figures 4I–4K). While the LIR of p62 forms three backbone hydrogen bonds with the β2-strand of LC3B, five bonds are formed between the CLIR and LC3C (Figures 4J and 4K), which may partly compensate for the missing aromatic-pocket interactions.

### Origin of LC3C-Specificity in NDP52: Acidic Flanking Sequences in LIR and CLIR

Stretches of acidic residues N-terminal of the canonical LIR β sheet contribute to ATG8-binding (Noda et al., 2010). In p62, interactions of acidic residues upstream of the LIR with basic residues in the α1 helix of LC3B are formed (Figure 4K). In NDP52, Asp132 and Glu130 compete for Lys55 of LC3C, while Glu128 does not participate in interactions. Asn129 interacts with Lys32 of the α2 helix of LC3C (Figure 4J, see below). The N-terminal α1 helix of LC3C is disordered in our structure; it is possible that this helix is stabilized by acidic residues upstream of canonical LIRs (Noda et al., 2010). However, the SKICH domain of NDP52 in our crystals likely imposes restraints on the CLIR-linker, which would be absent from flexible peptides that have been used previously to study LIR interactions.

### The Unoccupied Aromatic Pocket

To quantify affinity and specificity of the CLIR interactions and to understand the contribution of the aromatic pocket to LC3 affinity (Figure 5A), FITC-labeled 14-mer peptides corresponding to the NDP52 CLIR region (aa 128–141) were used to determine binding constants (*K*_*D*_) by fluorescence anisotropy equilibrium measurements. The CLIR peptide bound LC3C with a *K*_*D*_ of 1.6 μM, while LC3A was bound with ∼10x lower affinity (15.1 μM) (Figures 5B and 5C). As a negative control, Val136 in the peptide was mutated to Ser, and, consistent with the LUMIER analysis (Figure 3D), the mutant CLIR peptide did not interact with LC3C or LC3A (Figures 5B and 5C).

To understand the relative contribution of the aromatic pocket to the binding of LIR and CLIR peptides, we replaced Ile133 in the CLIR peptide with Trp. LC3C bound CLIR^I133W^ with 14-fold higher affinity than the CLIR^WT^ peptide, resulting in the highest affinity LC3 interaction reported so far (*K*_*D*_ = 110 nM) (Figure 5B). Importantly, the CLIR^I133W^ peptide also bound LC3A with high affinity (290 nM), which represents a > 50-fold improvement over CLIR^WT^ (Figure 5C). However, the specificity of NDP52 for LC3C over LC3A, defined as the ratio of *K*_*D*_ values, diminished from 10-fold to 3-fold (Figure 5C). To test whether the mutation confers a similar phenotype to full-length NDP52, we performed a LUMIER binding assay. In contrast to the preferential interaction of wild-type NDP52 with LC3C, we observed strong and nonselective binding of NDP52^I133W^ to all human ATG8 orthologs (Figure 5D). These data show that the NDP52 CLIR motif comprising a Leu-Val-Val sequence is able to distinguish efficiently between LC3C and its paralogs, and that mutation of the CLIR to utilize the aromatic pocket (CLIR^I133W^) enhances affinity but reduces the specificity for LC3C.

To investigate in vivo whether the enhanced affinity of NDP52^I133W^ has functional consequences for autophagy, we depleted HeLa cells of LC3C and monitored the recruitment of LC3B to *S.* Typhimurium (Figure 5E). Cells lacking LC3C did not efficiently recruit LC3B to bacteria, as we had observed earlier (Figure 2E). Expression of NDP52^I133W^, but not wild-type NDP52, enabled the targeting of bacteria by LC3B in the absence of LC3C, indicating that the enhanced affinity of NDP52^I133W^ provides novel functionality to cells.

### Subtle Differences in LC3 Isoforms Enable Specific Interactions with Autophagy Receptors

We next wanted to understand the structural features of LC3C that allow specific recognition by the NDP52 CLIR. However, despite the clear ligand specificity of NDP52, the residues of LC3C lining the CLIR-binding pocket are highly conserved among ATG8 orthologs. This conservation was to be expected since canonical LIRs bind all Atg8-family members, including LC3C.

**Figure 6:**
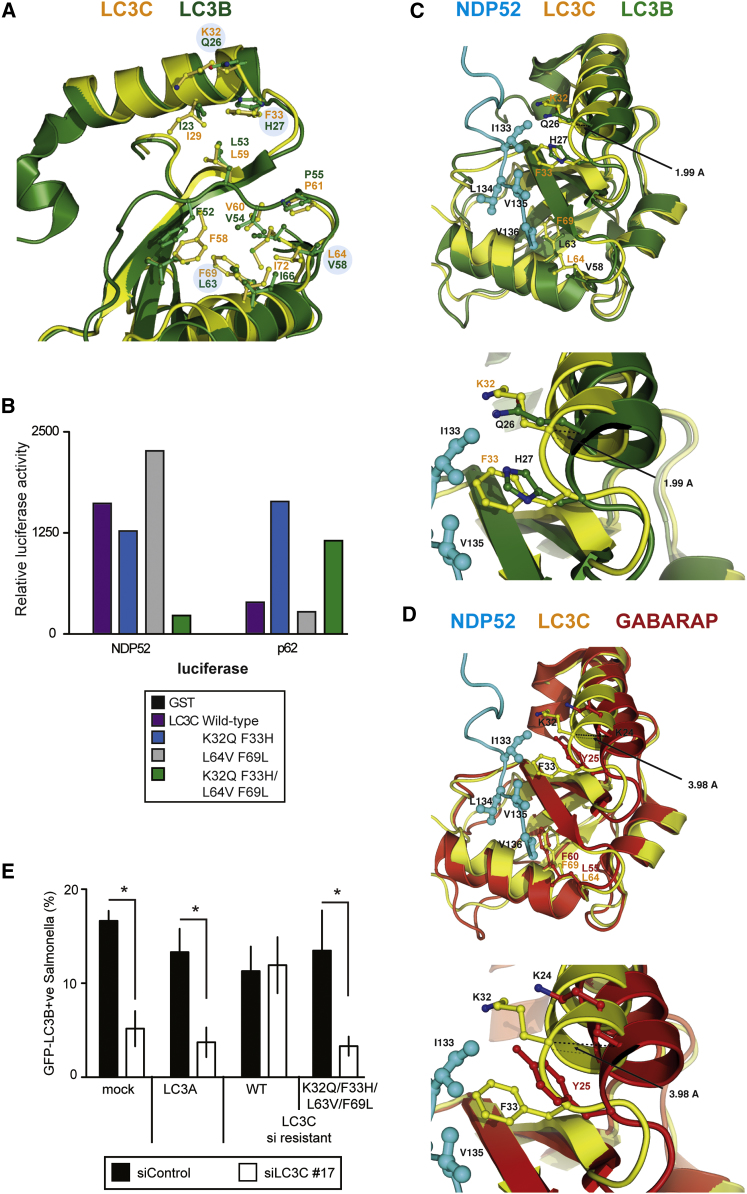
Residues in LC3C Providing Specificity for NDP52 (A) Superposition of LC3C (yellow) and LC3B (green), with residues lining the CLIR interaction groove in ball-and-stick representation. (B) LUMIER binding assay. Normalized ratio between luciferase activity bound to beads and present in lysates. Lysates of 293ET cells expressing the indicated luciferase fusion constructs, incubated with purified GST-tagged proteins bound to beads. (C–D) Superposition of NDP52 (blue), LC3C (yellow), LC3B (green) and GABARAP (red) with residues mediating the interaction in ball-and-stick representation. Enlarged: movement of the α2-helix in LC3B and GABARAP compared to LC3C. (E) Percentage of bacteria coated by LC3B in HeLa cells stably expressing GFP-tagged LC3B. Cells transduced with mock, LC3A or the indicated LC3C alleles and treated with siRNA against LC3C were infected with *S*. Typhimurium for 1 hr. LC3A and LC3C mRNA levels are further characterized in Figure S6C. (All panels) Mean and s.d. of triplicate cultures. ^∗^p < 0.05, Student's t test.

Nevertheless, close inspection of the CLIR-binding site and comparison with LC3A/B identified a number of subtle changes in two regions involved in CLIR interactions (Figures 6A and S1A). First, two residues in the Leu-pocket of LC3C have bulkier side chains compared to LC3A/B. Val58 and Leu63 in LC3B are replaced by Leu64 and Phe69 in LC3C, respectively. These changes reduce the size of the Leu-pocket to generate the hydrophobic surface specifically suited for interaction with the flat hydrophobic patch of the CLIR. Second, Gln26 and His27 of the LC3B α2 helix are replaced by Lys32 and Phe33 in LC3C, respectively (Figure 6A). Hydrophobic interactions between LC3C Phe33 and CLIR Val135 can therefore contribute to specificity.

The LIR docking site in GABARAP subfamily members is even more closely related to LC3C than the binding sites in LC3A and LC3B. Most residues that differ between LC3C and LC3A/B, i.e., Lys32, Leu64, and Phe69 in LC3C, are evolutionary conserved in GABARAP subfamily members. Only the residue corresponding to Phe33 in LC3C is replaced by Tyr in GABARAP subfamily members (Figure S1A). However, the superposition of structures for LC3A, LC3B, LC3C, GABARAP, and GABARAPL1 revealed that the α2-helix in GABARAP subfamily members is reoriented such that the Lys32- and Phe33-equivalent side chains may not be accessible for NDP52 (Figures 6C, 6D, S6A, and S6B).

Based on these considerations we attempted to mutate LC3C so that it would no longer interact with NDP52, but retain its LIR binding properties. Double mutations of LC3C to corresponding residues in LC3B (i.e., L64V/F69L or K32Q/F33H) were insufficient to inhibit NDP52 binding (Figure 6B). Binding of the mutant LC3C to NDP52 was abrogated only when all four mutations were introduced. Importantly, all analyzed LC3C mutants still bound the nonselective LIR of p62 with similar or even better affinity than wild-type LC3C. The specificity of the LC3C-NDP52 interaction is therefore determined by the combination of at least four residues (Lys32, Phe33, Leu64, Phe69) in the periphery of the CLIR binding pocket of LC3C, which contribute positively to the binding of NDP52 while disfavoring interactions with p62. Complementation of cells lacking LC3C with LC3A or siRNA-resistant LC3C alleles revealed that only wild-type LC3C but not LC3A or LC3C K32Q/F33H/L64V/F69L, which is specifically impaired in binding to NDP52, re-established antibacterial autophagy as detected by the recruitment of LC3B to *S.* Typhimurium (Figures 6E and S6C). We therefore conclude that the binding of NDP52 to LC3C is an essential requirement for antibacterial autophagy that p62 cannot fulfill.

## Discussion

In this study we discovered that the ubiquitin-binding autophagy receptor NDP52 binds preferentially to LC3C, in contrast to other autophagy adaptors including p62 and NBR1 that nonselectively interact with several human ATG8 orthologs. The specificity of NDP52 for LC3C results from a noncanonical LIR in NDP52 that fails to occupy the aromatic pocket of the LIR-docking site in LC3C and rather contacts several LC3C-specific residues in its periphery. Cells lacking LC3C, similar to cells lacking NDP52, fail to restrict the growth of *S.* Typhimurium in their cytosol, because in the absence of either protein cytosolic bacteria are not taken up efficiently into autophagosomes. Cells lacking wild-type LC3C but expressing LC3C K32Q/F33H/L64V/F69L, which is selectively impaired in its ability to bind NDP52 while still binding to p62, similarly fail to execute antibacterial autophagy. LC3C hence serves an essential role in antibacterial autophagy by providing a binding site for the cargo-selecting autophagy receptor NDP52 on the autophagosomal membrane. As far as we know, this is the first nonredundant function of an ATG8 ortholog. Our data suggest that the ATG8 orthologs implement an additional layer of specificity in the autophagy system.

Several autophagy genes that exist as single copies in yeast comprise gene families in complex organisms. The human genome, for example, encodes several orthologs of ATG4, ATG8, ATG9, and ATG16. Among the ATG8 homologs, the LC3 subfamily has been suggested to initiate phagophore expansion, while the GABARAP subfamily functions further downstream in autophagosome biogenesis (Weidberg et al., 2010). Notwithstanding this functional specialization between the LC3 and GABARAP subfamilies, the ability of evolutionary distant ATG8 orthologs to complement ATG8-deficient yeast suggests that core ATG8 functions have been maintained during evolution (Ketelaar et al., 2004; Williams et al., 2009; Alberti et al., 2010; Pérez-Pérez et al., 2010). The selective binding of LC3C to NDP52 and their role in restricting the proliferation of cytosolic *S.* Typhimurium are therefore reflecting a specialized nonredundant function of LC3C, namely the autophagy of NDP52-selected cargo.

The fact that NDP52 and LC3C are required to recruit other ATG8 family members to *S.* Typhimurium suggests a hierarchy among ATG8 orthologs. Within this hierarchy the NDP52-LC3C ligand pair may mediate the recruitment of phagophores and/or membranes to expand phagophores that are precharged with multiple ATG8 orthologs. The size of antibacterial autophagosomes, which are significantly larger than conventional autophagosomes, may require such a dedicated mechanism of membrane delivery, for example to outcompete other membrane-demanding processes. Alternatively, membranes for antibacterial autophagy may be selectively recruited from one of several sources that supply membranes for autophagy (Hayashi-Nishino et al., 2009; Ylä-Anttila et al., 2009; Hailey et al., 2010; Ravikumar et al., 2010; van der Vaart et al., 2010; Yen et al., 2010). Considering the multilamellar membrane structure of antibacterial autophagosomes (Zheng et al., 2009; Kageyama et al., 2011) it seems also possible that the NDP52-LC3C ligand pair is specifically required for the generation of this unusual autophagosomal structure. In addition to a role in phagosome biogenesis, the specific binding of NDP52 to LC3C could enable the preferential handling of NDP52-selected autophagosomal content. During antibacterial autophagy this may involve the activation of effector mechanisms to kill the infectious cargo and the generation of antigenic peptides for presentation to T-lymphocytes. Although we do not know how many autophagy receptors exist that distinguish between ATG8 orthologs, a number of proteins bind preferentially to individual ATG8 orthologs in vivo (Behrends et al., 2010). These data suggest that the selective handling of autophagy cargo may not be limited to antibacterial autophagy. Intriguingly, the mitophagy adaptor Nix, binds LC3A but not LC3B (LC3C was not tested) (Novak et al., 2010). Nix also binds members of the GABARAP subfamily, but since these function downstream of the LC3 subfamily (Weidberg et al., 2010) efficient mitophagy may depend on the ability of Nix to distinguish between LC3 subfamily members in order to form autophagosomes large enough to engulf mitochondria.

The specificity of NDP52 for LC3C is mediated by a noncanonical LIR comprising the tripeptide Leu-Val-Val, which we refer to as the CLIR motif. This binding site in NDP52, located between the SKICH domain and a coiled-coil forming region, is distinct from a previously predicted LIR within the SKICH domain, i.e., Trp-Ile-Gly-Ile^55-58^ (Kraft et al., 2010). While this sequence is reminiscent of a LIR motif, our structure reveals it to be buried in a β sandwich-fold and inaccessible for canonical ATG8 interactions (Figure 4A). Furthermore, mutations in the CLIR motif of NDP52 abrogate interactions with LC3C (Figures 3A and 3D).

The CLIR-LC3C binding mode is reminiscent of canonical LIR-LC3 interactions since both rely on the formation of an intermolecular β sheet between the CLIR/LIR peptide and the β2-strand of the ATG8 ortholog (Noda et al., 2008). In addition, the side chains of CLIR and LIR peptides establish extensive hydrophobic contacts to their ligands. A major difference between CLIR and LIR peptides is the lack of an aromatic residue in the former, resulting in curtailed affinity of NDP52 for all tested ATG8 orthologs including its preferred ligand LC3C. However, the corresponding aromatic pocket is present in all ATG8 orthologs, including LC3C, which explains why LC3C can be bound also by LIR-containing autophagy receptors. Despite lacking an aromatic residue, the CLIR peptide binds LC3C with a *K*_*D*_ of 1.6 μM, an affinity similar to that of canonical LIRs (Noda et al., 2008; Rozenknop et al., 2011). It therefore appears that the loss in affinity due to the unoccupied aromatic pocket in LC3C is compensated by additional hydrophobic contacts of the Leu-Val-Val motif in the CLIR β strand. These contacts require a complementary binding site in LC3C, defined by Lys32, Phe33, Leu64 and Phe69 that other human ATG8 orthologs lack because contact residues are either nonconserved or misaligned. The specificity of NDP52 for LC3C is therefore due to the lack of an aromatic residue in the CLIR β strand and the compensatory acquisition of a Leu-Val-Val binding site in LC3C.

Further experiments are required to test whether additional autophagy receptors with selective specificity for LC3C or other ATG8 orthologs exist, whether they use similar or unrelated mechanism to achieve binding specificity, and whether they perform essential functions that extend to their preferential binding partner.

## Experimental Procedures

### Plasmids

M5P or derived plasmids were used to express proteins in mammalian cells (Randow and Sale, 2006). ORFs were amplified by PCR or have been described (Bloor et al., 2008, 2010; Thurston et al., 2009).

### Bacteria

Infections with *S*. Typhimurium 12023 and *S. flexneri* M90T were performed as described previously (Thurston et al., 2009).

### RNAi

Experiments were performed 72 hr after transfection with siRNAs.

### Immunoprecipitation, Western Blot, and LUMIER Assay

These were performed as described previously (Barrios-Rodiles et al., 2005; Ryzhakov and Randow, 2007).

### Microscopy

HeLa cells, grown on glass coverslips and fixed in 4% paraformaldehyde were incubated with primary, followed by secondary antibodies before being mounted. Confocal images were taken with a x63, 1.4 numerical aperture objective on either a Zeiss 710 or a Zeiss 780 microscope.

### Protein Expression

Proteins were expressed in *E. coli* using pETM30 vectors for biochemical experiments and pOPIN-K for structural studies. For NMR experiments bacteria were grown in M9 minimal medium supplemented with ^15^N ammonium chloride and/or ^13^C glucose as required.

### NMR

Standard triple resonance experiments (HNCACB, HNCA, CBCA(CO)NH, HNCACO and HNCO) were used to assign backbone resonances of ^13^C,^15^N-labeled NDP52 (21–141) at a sample concentration of 150 μM.

### Crystal Structure Analysis

Crystallization conditions were screened by the vapor diffusion method. The structure of NDP52 SKICH domain was determined by single anomalous dispersion methods. The structure of the NDP52-LC3C complex was solved by molecular replacement using the NDP52 SKICH domain and LC3B as search models.

### Fluorescence Anisotropy

Fluorescent measurements of purified ATG8 orthologs, serially diluted and mixed with equal volumes of 100 nM FITC-labeled NDP52 peptides, were performed on a PheraStar plate reader.

## Figures and Tables

**Table 1:** Pairwise Identities/Similarities of Human ATG8 Orthologs, Ubiquitin, and NEDD8 in Percent

	LC3B	LC3C	GABARAP	GABARAPL1	GABARAPL2	Ubiquitin	NEDD8
**LC3A**	**83**/93	**57**/71	**31**/56	**33**/58	**40**/64	**12**/28	**15**/32
**LC3B**		**53**/69	**31**/58	**32**/59	**38**/64	**18**/33	**15**/31
**LC3C**			**38**/62	**39**/60	**40**/61	**11**/26	**15**/26
**GABARAP**				**87**/95	**58**/77	**16**/25	**12**/22
**GABARAPL1**					**61**/76	**14**/27	**11**/26
**GABARAPL2**						**15**/34	**17**/31
**Ubiquitin**							**58**/78

**Table 2:** Crystallization Data Collection and Refinement Statistics

	NDP52_Apo (SAD)	NDP52_Apo	NDP52/LC3C
**Data Collection Statistics**			

Beamline		ESRF ID29	ESRF ID14-4
Wavelength (Å)	1.5418	1.0000	0.9395
Space Group	*P*4_1_2_1_2	*P*2_1_2_1_2_1_	*C*2
Unit Cell (Å)	*a, b* = 75.94 *c* = 94.25	*a* = 36.61 *b* = 37.46 *c* = 90.40	*a* = 195.72 *b* = 37.42 *c* = 40.28 **β** = 94.66
Resolution (Å)	59.13-2.70 (2.85-2.70)	26.18-1.35 (1.42-1.35)	48.77-2.50 (2.64-2.50)
Observed reflections	108269 (15299)	95964 (13893)	41026 (5995)
Unique reflections	8055 (1126)	27864 (4013)	10306 (1472)
Redundancy	13.4 (13.6)	3.4 (3.5)	4.0 (4.1)
Completeness (%)	100.0 (100.0)	99.3 (99.9)	99.8 (100.0)
*R*_merge_	0.115 (0.595)	0.064 (0.559)	0.109 (0.446)
<I/σI >	21.1 (4.4)	10.6 (2.8)	9.4 (3.2)

**Phasing Statistics**			

FOM	0.235		
FOM after DM	0.899		

**Refinement Statistics**			

Reflections in test set		1361	487
*R*_cryst_		15.5	21.0
*R*_free_		18.9	25.2

**Number of Groups**			

Protein residues		93	218
Ions and ligand atoms		0	0
Water		178	41
Wilson B-factor		12.4	44.6

**RMSD from Ideal Geometry**			

Bond length (Å)		0.01	0.008
Bond angles (°)		1.272	1.046

**Ramachandran Plot Statistics**			

In Favored Regions (%)		93 (100)	212 (97.25)
In Allowed Regions (%)		0 (0.00)	5 (2.29)
Outliers (%)		0 (1.03)	1 (0.46)

Values in parentheses are for the highest resolution shell.

## Accession Numbers

Protein Data Bank accession numbers for NDP52 and the NDP52 LC3C complex are 3VVV and 3VVW, respectively.

## Supplemental Information

Document S1. Six Figures, Supplemental Experimental Procedures, and Supplemental References
