# Supplementary material for: LC3C, Bound Selectively by a Noncanonical LIR Motif in NDP52, Is Required for Antibacterial Autophagy
Source: Mol Cell. 2012 Nov 9;48(3):329–42. doi: 10.1016/j.molcel.2012.08.024 (PMC3510444; doi:10.1016/j.molcel.2012.08.024)
Supplement: Document S1. Six Figures, Supplemental Experimental Procedures, and Supplemental References [file mmc1.pdf]

Molecular Cell, Volume *48*

Supplemental Information

LC3C, Bound Selectively by a Noncanonical LIR Motif in NDP52, Is Required for Antibacterial Autophagy

Natalia von Muhlinen, Masato Akutsu, Benjamin J. Ravenhill, Ágnes Foeglein, Stuart Bloor, Trevor J. Rutherford, Stefan M.V. Freund, David Komander, and Felix Randow

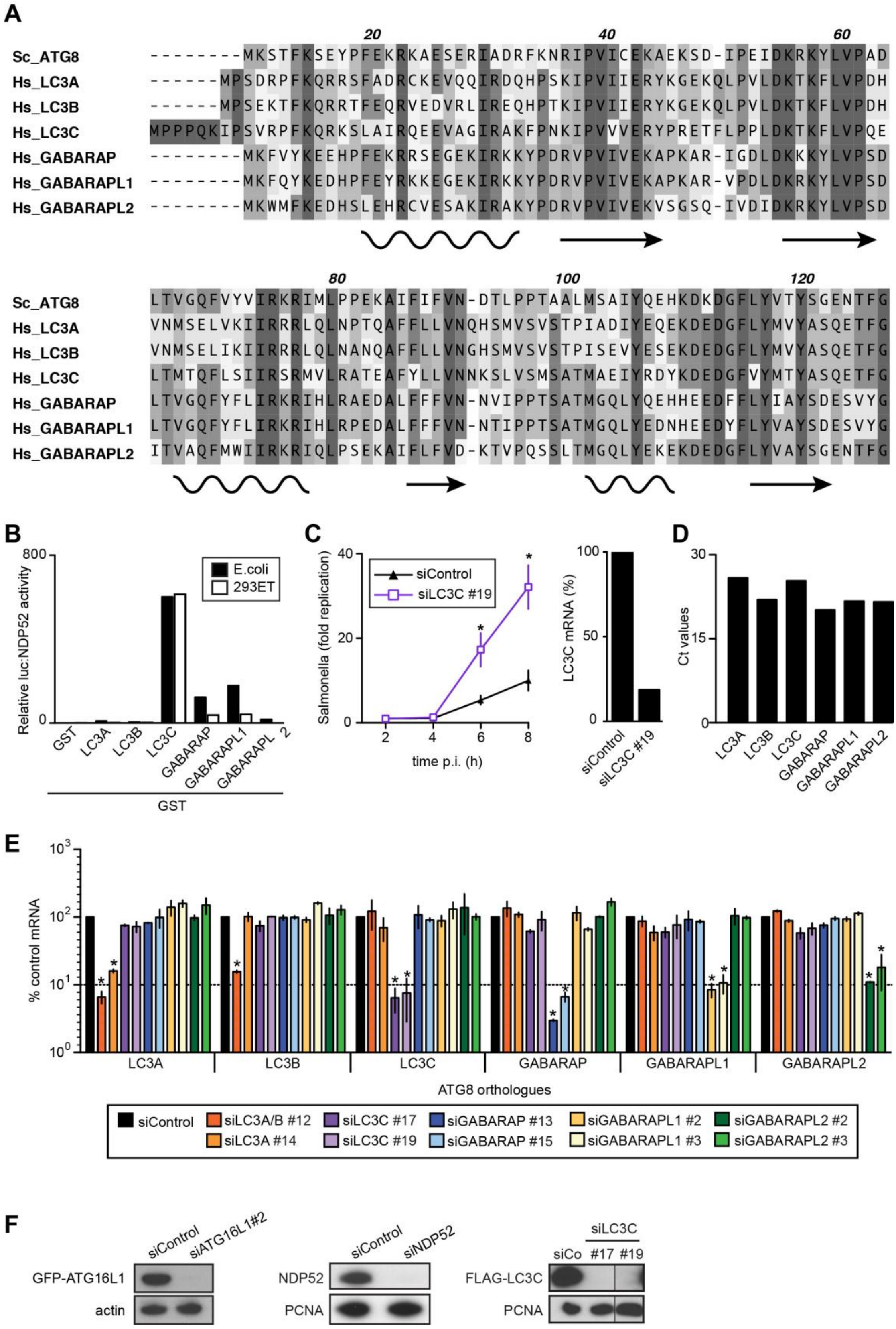

A
20
40
60
Sc_ATG8 --------MKSTFKSEYPFEKRKAESERIADRFKNRIPVICEKAEKSD-IPEIDKRKYLVPAD
Hs_LC3A ------MPSDRPFKQRRSFADRCKEVQQIRDQHPSKIPVIIERYKGEKQLPVLDKTKFLVPDH
Hs_LC3B ------MPSEKTFKQRRTFEQRVEDVRLIREQHPTKIPVIIERYKGEKQLPVLDKTKFLVPDH
Hs_LC3C MPPPQKIPSVRPFKQRKSLAIRQEEVAGIRAKFPNKIPVVVERYPRETFLPPLDKTKFLVPQE
Hs_GABARAP --------MKFVYKEEHPFEKRRSEGEKIRKKYPDRVPVIVEKAPKAR-IGDLDKKKYLVPSD
Hs_GABARAPL1 --------MKFQYKEDHPFEYRKKEGEKIRKKYPDRVPVIVEKAPKAR-VPDLDKRKYLVPSD
Hs_GABARAPL2 --------MKWMFKEDHSLEHRCVESAKIRAKYPDRVPVIVEKVSGSQ-IVDIDKRKYLVPSD
80
100
120
Sc_ATG8 LTVGQFVYVIRKRIMLPPEKAIFIFVN-DTLPPTAALMSAIYQEHKDKDGFLYVTYSGENTFG
Hs_LC3A VNMSELVKIIRRRLQLNPTQAFFLLVNQHSMVSVSTPIADIYEQEKDEDGFLYMVYASQETFG
Hs_LC3B VNMSELIKIIRRRLQLNANQAFFLLVNGHSMVSVSTPISEVYESEKDEDGFLYMVYASQETFG
Hs_LC3C LTMTQFLSIIRSRMVLRATEAFYLLVNNKSLVSMSATMAEIYRDYKDEDGFVYMTYASQETFG
Hs_GABARAP LTVGQFYFLIRKRIHLRAEDALFFFVN-NVIPPTSATMGQLYQEHHEEDFFLYIAYSDESVYG
Hs_GABARAPL1 LTVGQFYFLIRKRIHLRPEDALFFFVN-NTIPPTSATMGQLYEDNHEEDYFLYVAYSDESVYG
Hs_GABARAPL2 ITVAQFMWIIRKRIQLPSEKAIFLFVD-KTVPQSSLTMGQLYEKEKDEDGFLYVAYSGENTFG
B
Relative luc:NDP52 activity
800
0
E.coli
293ET
GST
LC3A
LC3B
LC3C
GABARAP
GABARAPL1
GABARAPL 2
GST
C
Salmonella (fold replication)
40
20
0
siControl
siLC3C #19
2
4
6
8
time p.i. (h)
LC3C mRNA (%)
100
50
0
siControl
siLC3C #19
D
Ct values
30
15
0
LC3A
LC3B
LC3C
GABARAP
GABARAPL1
GABARAPL2
E
% control mRNA
10^3
10^2
10^1
10^0
LC3A
LC3B
LC3C
GABARAP
GABARAPL1
GABARAPL2
ATG8 orthologues
siControl
siLC3A/B #12
siLC3A #14
siLC3C #17
siLC3C #19
siGABARAP #13
siGABARAP #15
siGABARAPL1 #2
siGABARAPL1 #3
siGABARAPL2 #2
siGABARAPL2 #3
F
siControl
siATG16L1#2
GFP-ATG16L1
actin
siControl
siNDP52
NDP52
PCNA
siLC3C
siCo
#17
#19
FLAG-LC3C
PCNA

Figure S1

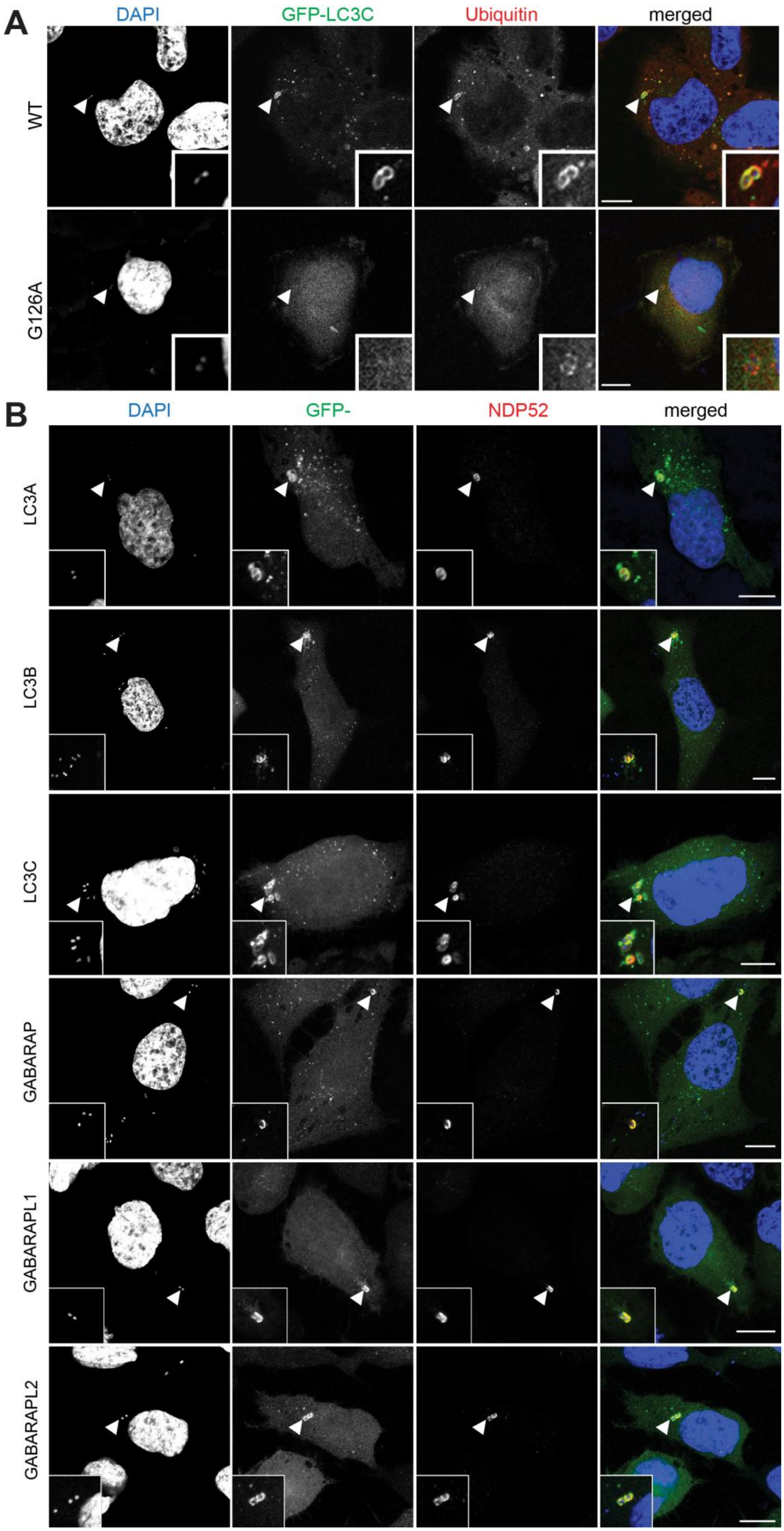
A
DAPI
GFP-LC3C
Ubiquitin
merged
WT
G126A
B
DAPI
GFP-
NDP52
merged
LC3A
LC3B
LC3C
GABARAP
GABARAPL1
GABARAPL2

Figure S2

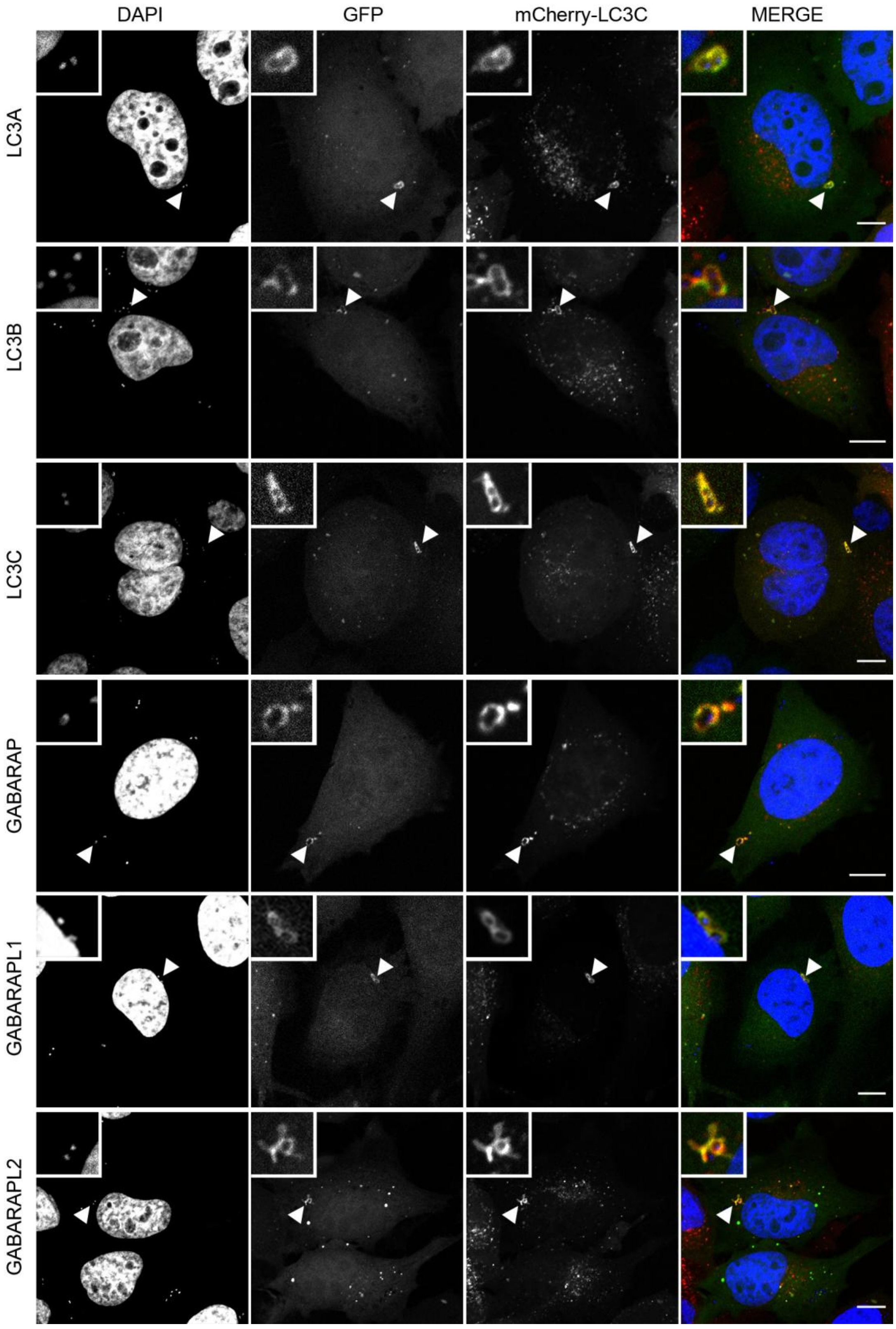
DAPI
GFP
mCherry-LC3C
MERGE
LC3A
LC3B
LC3C
GABARAP
GABARAPL1
GABARAPL2

Figure S3

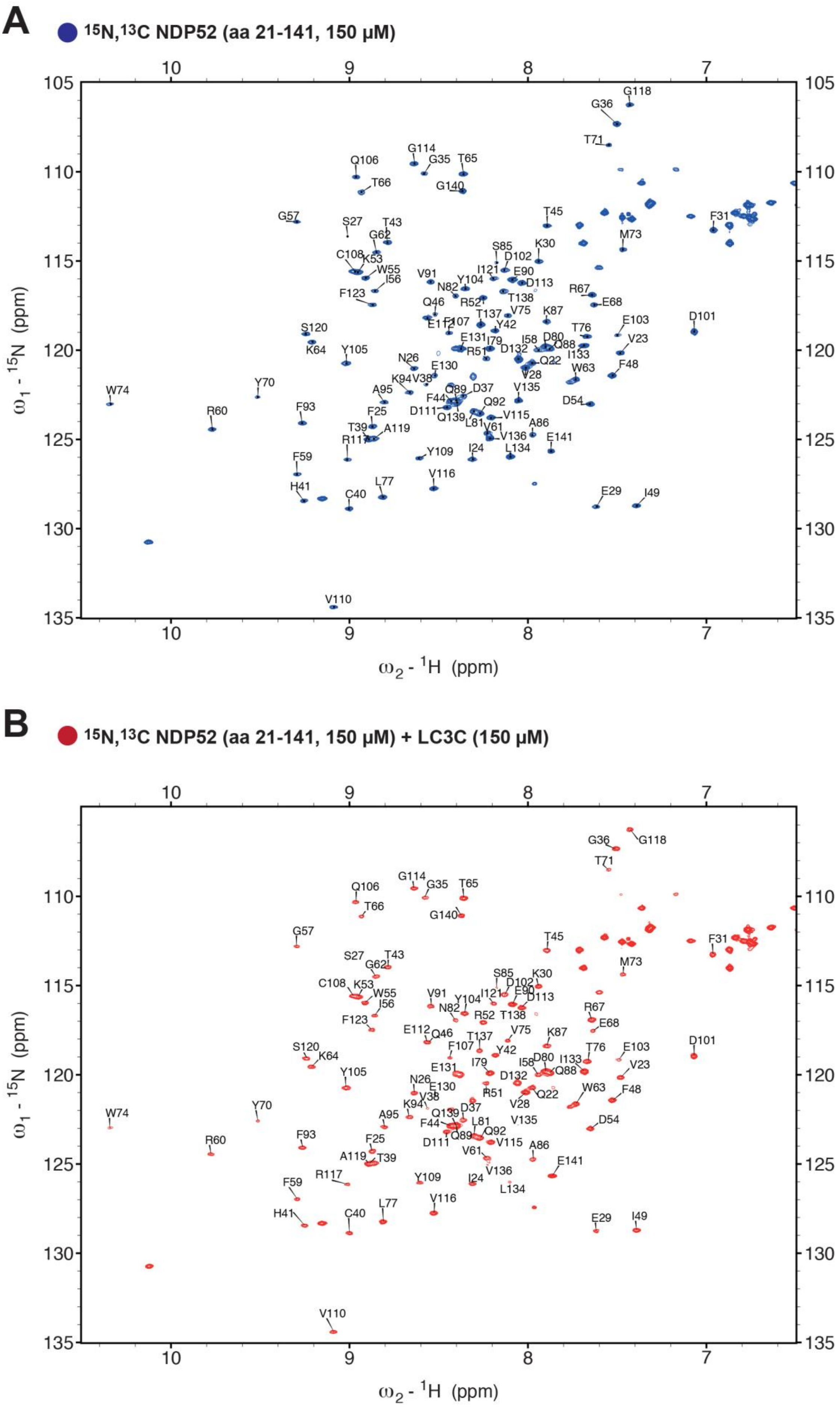
A
15N,13C NDP52 (aa 21-141, 150 μM)
ω1 - 15N (ppm)
ω2 - 1H (ppm)
10
9
8
7
105
110
115
120
125
130
135
G118
G36
T71
G114
Q106
G35
T65
T66
G140
G57
S27
T43
T45
F31
G62
M73
S85
K30
D102
C108
K53
W55
I56
V91
I121
E90
N82
Y104
D113
R67
F123
R52
T138
E68
Q46
T137
V75
K87
E112
F107
Y42
S120
E103
D101
T76
K64
E131
I79
I58
D80
Q88
V23
Y105
R51
D132
I133
N26
Q22
F48
E130
W63
V28
K94
V38
A95
Q89
D37
V135
W74
Y70
F44
D54
Q92
F93
R60
D111
Q139
V115
F25
L81
A86
T39
V61
A119
R117
V136
E141
L134
I24
Y109
F59
V116
L77
H41
E29
I49
C40
V110
B
15N,13C NDP52 (aa 21-141, 150 μM) + LC3C (150 μM)

Figure S4

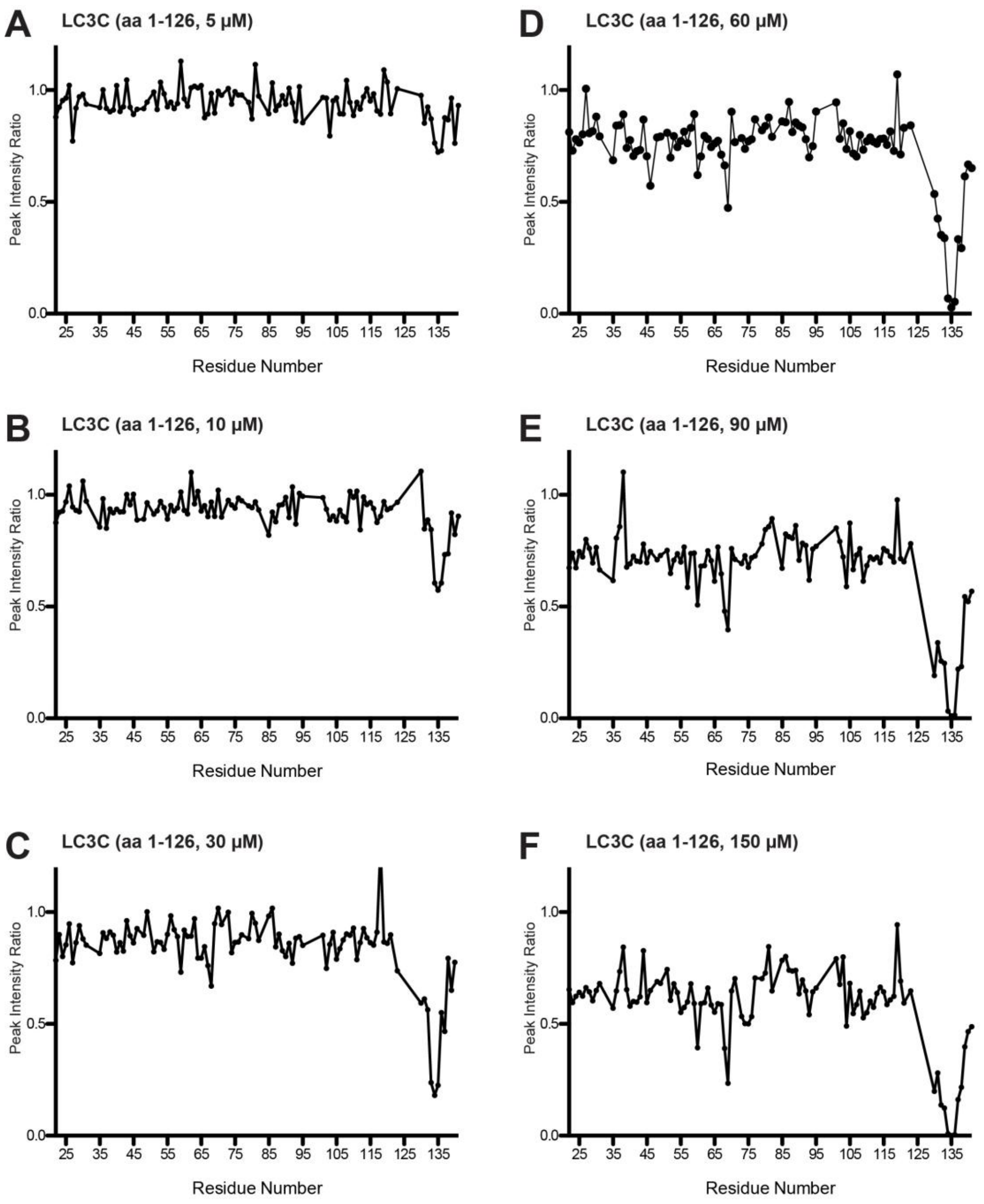
A
LC3C (aa 1-126, 5 μM)
B
LC3C (aa 1-126, 10 μM)
C
LC3C (aa 1-126, 30 μM)
D
LC3C (aa 1-126, 60 μM)
E
LC3C (aa 1-126, 90 μM)
F
LC3C (aa 1-126, 150 μM)
Peak Intensity Ratio
Residue Number
0.0
0.5
1.0
25 35 45 55 65 75 85 95 105 115 125 135

Figure S5

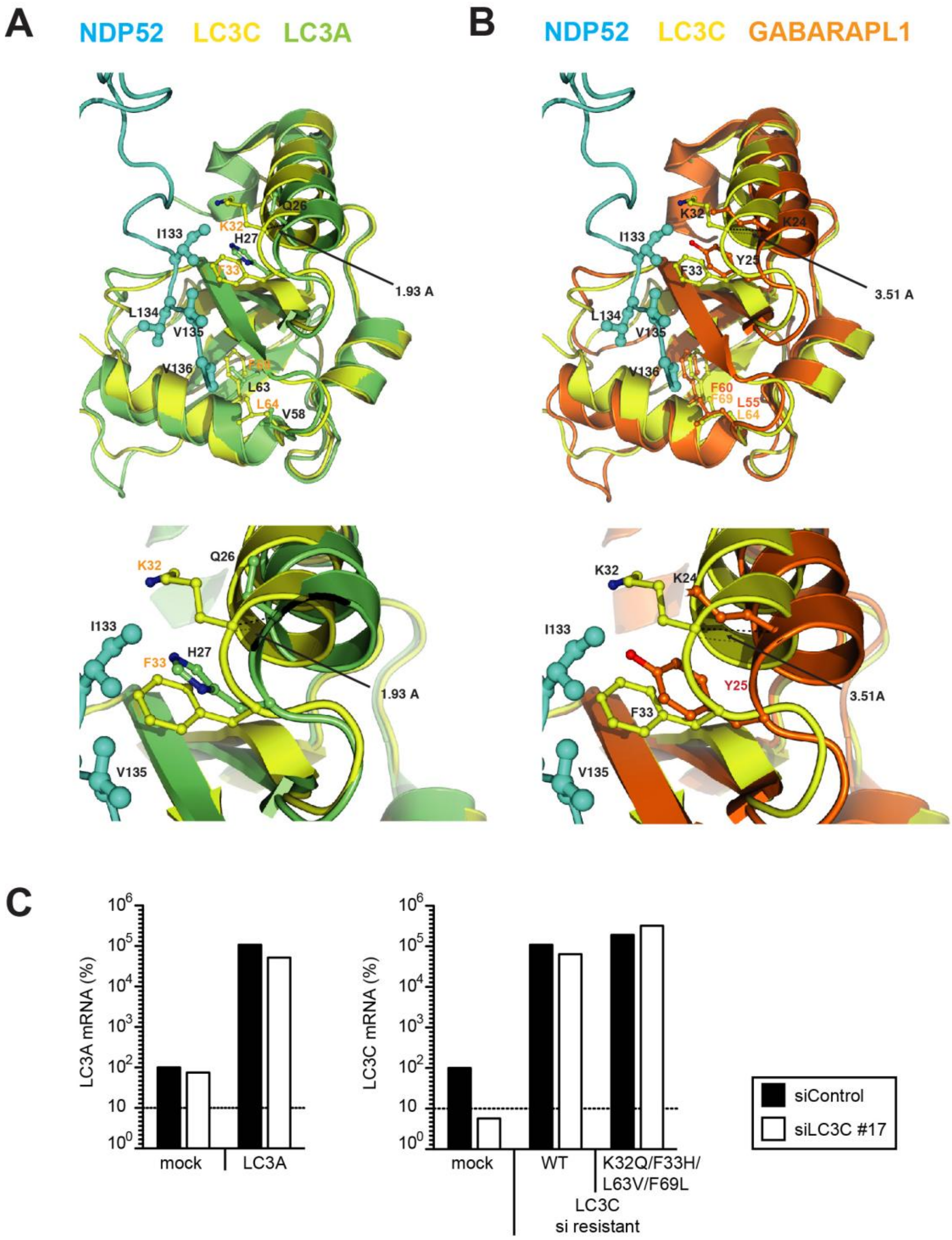
A
NDP52 LC3C LC3A
Q26
K32
I133
H27
F33
1.93 A
L134
V135
V136
L63
L64
V58
B
NDP52 LC3C GABARAPL1
K32
K24
I133
Y25
F33
3.51 A
L134
V135
V136
F60
F69
L55
L64
3.51A
C
LC3A mRNA (%)
LC3C mRNA (%)
mock
LC3A
WT
K32Q/F33H/
L63V/F69L
LC3C
si resistant
siControl
siLC3C #17

Figures S6

Supplemental Figure Legends

Figure S1, related to Figure 1

A) Alignment of ATG8 family members from S. cerevisiae (Sc) and Homo sapiens (Hs). Mature proteins with C-terminally exposed Gly are shown. α-helices and β-strands are indicated by waved lines and arrows, respectively.

B) LUMIER binding assay. Normalized ratio between luciferase activity bound to beads and present in lysates. Lysates of 293ET cells or *E. coli* expressing NDP52 fused to luciferase, incubated with purified GST-tagged ATG8 orthologs bound to beads.

C) Left: Kinetics of *S.* Typhimurium replication in 293ET cells transfected with the indicated siRNAs. Bacteria counted on the basis of their ability to form colonies on agar plates. Right: LC3C mRNA levels in 293ET cells treated with the indicated siRNAs as determined by quantitative PCR.

D) mRNA levels of ATG8 orthologs in HeLa cells determined by quantitative PCR. Ct cycle threshold

E) Normalized mRNA levels of ATG8 orthologs in HeLa cells transfected with the indicated siRNAs as determined by quantitative PCR.

F) Lysates of HeLa cells expressing GFP-tagged ATG16L1, endogenous NDP52 or FLAG-tagged LC3C, treated with the indicated siRNAs and probed three days after transfection with the indicated antibody.

Figure S2, related to Figure 2

A-B) Microphotographs of HeLa cells stably expressing the indicated GFP-tagged ATG8 orthologs, infected with *S.* Typhimurium for 1 h, and stained with DAPI and an antibody against ubiquitin (**A**) or NDP52 (**B**).

Scale bar 10 μm.

Figure S3, related to Figure 2

Microphotographs of HeLa cells stably expressing the indicated GFP-tagged ATG8 orthologs and mCherry-LC3C, infected with *S.* Typhimurium for 1 h, and stained with DAPI.

Scale bar 10 μm.

Figure S4, related to Figure 3

A) Assigned $^{15}N,^{1}H$-HSQC spectra of ^{15}N-labeled NDP52 (aa 21-141) at 150 μM concentration. Assignments wee obtained from triple resonance experiments using $^{15}N,^{13}C$-labeled NDP52 (aa 21-141) at 150 μM concentration.

B) Assigned ^{15}N-HSQC spectra of ^{15}N-labeled NDP52 (aa 21-141, 150 μM) in presence of 150 μM LC3C (aa 1-126). A section of the overlay of spectra in **A** and **B** is shown in **Fig. 3B**.

Figure S5, related to Figure 3

(A-F) Relative exchange broadening by NDP52 residue number, comparing the intensity ratio of NDP52 (aa 21-141, 150 μM) in absence and presence of LC3C at **(A)** 5 μM, **(B)** 10 μM, **(C)** 30 μM, **(D)** 60 μM, **(E)** 90 μM and **(F)** 150 μM concentration.

Figure S6, related to Figure 6

A-B) Superposition of NDP52 (blue), LC3C (yellow), LC3A (green) and GABARAPL1 (orange) with residues mediating the interaction in ball-and-stick representation. Enlarged: movement of the α2-helix in LC3A and GABARAPL1 compared to LC3C.

C) Quantification of LC3A and LC3C mRNA in HeLa cells, either mock-transduced or stably expressing LC3A or siRNA-resistant LC3C alleles, upon transfection with the indicated siRNAs.

Supplemental Experimental Procedures

Antibodies. Antibodies were from Developmental Studies (LAMP1), BD Transduction Laboratories (p62), Abcam (NDP52, for western blots), Sigma (FLAG M2), Dabco (HRP-conjugated reagents), Chemicon (anti-*Renilla* luciferase), Santa Cruz (anti-PCNA), R&D Systems (Galectin-8) and Invitrogen (Alexa-conjugated anti-mouse and anti-rabbit antisera). The anti-NDP52 antiserum used for immunofluorescence was a kind gift of John Kendrick-Jones.

Plasmids. M5P or closely related plasmids were used to produce recombinant MLV for the expression of proteins in mammalian cells (Randow and Sale, 2006). pETM plasmids were gifts from A. Geerlof. Open reading frames encoding human ATG8 orthologs, NDP52, p62, NBR1, were amplified by PCR or have been described (Thurston et al., 2009; Bloor et al., 2010; 2008). Mutations were generated by PCR and verified by sequencing.

Bacteria. *S*. Typhimurium (strain 12023), kindly provided by David Holden, was grown overnight in LB and sub-cultured (1:33) in fresh LB for 3.5 h prior to infection. HeLa cells in 24-well plates were infected with 20 μl of such cultures for 15 min. Following two washes with warm phosphate buffered saline (PBS, pH 7.4) and incubation with 100 μg/ml gentamycin for 2 h, cells were cultured in 20 μg/ml gentamycin. To measure intracellular bacterial growth, cells from triplicate wells were lysed in 1 ml cold PBS containing 0.1 % Triton X100. Serial dilutions were plated in duplicate on TYE agar.

S. flexneri M90T, kindly provided by Chris Tang, was grown overnight in Tryptic Soy Broth (TSB) and sub-cultured (1:100) in fresh TSB until an OD of approximately 0.3 was reached. Bacteria were resuspended in warm IMDM and 100 μl were added to HeLa cells in 24-well plates. Samples were centrifuged for 10 min at 2000 rpm. Following an incubation at 37 °C for 30 min, cells were washed with warm PBS and cultured in 100 μg/ml gentamycin for 2 h and 20 μg/ml thereafter.

Cell culture. Cells were grown in IMDM supplemented with 10% FCS at 37°C in 5% CO2. HeLa cells were obtained from the European Collection of Cell Cultures.

RNAi. $5x10^4$ cells per well were seeded in 24-well plates. The following day cells were transfected with 40 pmol of siRNA (Invitrogen) using Lipofectamine 2000 (Invitrogen) in Optimem medium (Invitrogen). Optimem was replaced with complete IMDM medium after 4 h and experiments were performed after 72 h. siRNAs targeted the following sequences:

siNDP52 5'UUCAGUUGAAGCAGCUCUGUCUCCC (Thurston et al., 2009)

siLC3C#17 5' GCUUGGCAAUCAGACAAGAGGAAGU

siLC3C#19 5'AGGACAGACCCUGCAAUCCUCUCUA

siLC3A/B#12 5'CGGACCAUGUCAACAUGAGCGAGUU

siLC3A#14 5'GGCUUCCUCUAUAUGGUCUACGCCU

siGABARAP#13 5'GAGGGCGAGAAGAUCCGAAAGAAAU

siGABARAP#15 5'ACAGUGACGAAAGUGUCUACGGUCU

siGABARAPL1#2 5'GAGGACAAUCAUGAGGAAGACUAUU

siGABARAPL1#3 5'GAGGACGCCUUAUUCUUCUUUGUCA

siGABARAPL2#2 5'GAUCUUCCUGUUUGUGGAUAAGACA

siGABARAPL2#3 5'GCCUAACUAUGGGACAGCUUUACGA

Quantitative PCR. Following total RNA isolation from cells using a RNAeasy kit (QIAGEN), cDNA was synthesized with the SuperScript™ III Reverse Transcriptase kit (Invitrogen) according to the manufacturer's instructions. Gene expression was quantified using a Power SYBR qPCR green kit (Applied Biosystems) along with specific primer pairs according to the manufacturers instructions. Relative amounts of cDNA were calculated based on a standard curve obtained for each pair of primers and normalized to actin cDNA levels in each sample. The following primers were used: 5'-CCTGGCACCCAGCACAAT-3' and 5'-GCCGATCCACACGGA GTACT-3' for *ACTB*, encoding actin (control); 5'-CAGCACCCCAGCAAAATC-3' and 5'-TCTTTCTCCTGCTCGTAGA-3' for *MAP1LC3A*, encoding LC3A; 5'-AAGAAGGCCTGATTAGCA-3' and 5'-AAGAAGGCCTGATTAGCA-3' for *MAP1LC3B*, encoding LC3B; 5'-CCCAAGCGTCAGACCCTTC-3' and 5'-GCTCCGGATGATGCTGAG-3' for encoding *MAP1LC3C*, LC3C; 5'-TGCCGGTGATAGTAGAAA-3' and 5'-GGTGTTCCTGGTACAGCT-3' for *GABARAP*,

encoding GABARAP; 5’-TGCCCTCTGACCTTACTG-3’ and 5’-AGTCTTCCTCATGATTGTC-3’ for *GABARAPL1/GEC1*, encoding GABARAPL1; 5’-TCGCTGGAACACAGATG-3’ and 5’-TGTCCCATAGTTAGGCTG-3’ for *GABARAPL2/GATE-16*, encoding GABARAPL2.

Immunoprecipitation and Western blot. Post-nuclear supernatants from 2 x 10^6 HeLa cells were obtained following lysis in 150 mM NaCl, 0.1% (v/v) Triton-X100, 20 mM Tris–HCl (pH 7.4), 5 mM EDTA and Complete protease inhibitors (Roche). Samples were separated on 4–12% denaturing Bis-Tris gels (Invitrogen). Visualization following immunoblotting was performed using ECL detection reagents (Amersham Bioscience).

LUMIER assay.(Ryzhakov and Randow, 2007; Barrios-Rodiles et al., 2005) Binding assays with pairs of putative interactors, one fused to luciferase and the other fused to GST or Flag, were performed in LUMIER lysis buffer (150 mM NaCl, 0.1% (v/v) Triton-X100, 20 mM Tris–HCl (pH 7.4), 5% (v/v) glycerol, 5 mM EDTA and Complete protease inhibitors (Roche)). GST-fusion proteins were immobilized on beads before incubation for 2 h with equal amounts of luciferase tagged binding partners, as determined by luciferase activity. After washing in lysis buffer, proteins were eluted with glutathione in Renilla lysis buffer (Promega). Relative luciferase activity represents the ratio of activity eluted from beads and present in lysates.

Microscopy. HeLa cells were grown on glass cover slips prior to infection. Following infections, cells were washed twice with warm PBS and fixed in 4% paraformaldehyde in PBS for 30 min. Cells were washed twice in PBS and then quenched and permeabilized with PBS (pH 7.4) containing 1 M glycine and 0.1% (v/v) Triton X100 for 30 min. Cells were blocked for 30 min in PBTB (PBS, 0.1% (v/v) Triton X100, 2% (w/v) BSA). Cover slips were incubated with primary, followed by secondary antibodies for 1 h in PBTB before being mounting in medium containing DAPI (Vector Laboratories). At least 100 events per slide were scored in quantitative assays. Confocal images were taken with a x63, 1.4 numerical aperture objective on either a Zeiss 710 or a Zeiss 780 microscope.

Protein purification. ATG8 orthologs were cloned into pETM30 vectors and expressed as GST fusion proteins in *E. coli* BL21. Cells were grown at 37 °C to an OD_{600} of 0.6, followed by induction with 0.5 mM isopropyl-β-d-thiogalactoside and further incubation at 16 °C for 16 h. Cells were lysed in lysis buffer (25mM Tris HCl, 300mM NaCl, 20% glycerol, 2mM EDTA, pH 7.4) using EmulsiFlex. Lysates were incubated with GST beads, washed with high-salt buffer (25 mM Tris-HCl, 500 mM NaCl, pH 8.0) followed by TEV cleavage buffer (HEPES 25 mM and NaCl 150 mM, pH 6.0). ATG8 orthologs were cleaved with TEV (100 μg/ml) overnight. Eluates were collected, TEV depleted using nickel beads (QIAGEN), and ATG8 orthologs further purified using gel filtration (HiLoad 16/60 Superdex 75) in 25 mM HEPES (pH 6.0) containing 150 mM NaCl.

Protein expression and purification for structural studies. Constructs comprising NDP52 (21-141) and LC3C (1-126) were cloned into pOPIN-K vector that adds a PreScission protease-cleavable N-terminal glutathione-S-transferase (GST) tag (Berrow et al., 2007), and transformed into *E. coli* Rosetta2 pLacI cells (Novagen). Cells were grown at 37°C to an OD_{600} of 0.6, followed by induction with 0.5 mM isopropyl-β-d-thiogalactoside and further incubation at 25 °C for 16 h before harvesting. Cells were lysed by sonication in lysis buffer (25 mM Tris, 200 mM NaCl, 5 mM DTT, pH 8.0), and the lysate was cleared by centrifugation (20000 x g, 4°C, 30 min). The cleared lysate was incubated with 2 mL (per L cell culture) glutathione-sepharose 4B (GE Life Sciences) for 1 h at 4 °C, collected in a gravity flow column, and washed with high salt buffer (25 mM Tris, 500 mM NaCl, 5 mM DTT, pH 8.5 (NDP52) or pH 6.5 (LC3C)) and low salt buffer (20 mM Tris, 50 mM NaCl, 5 mM DTT, pH 8.5 (NDP52) or pH 6.5 (LC3C)). GST fusion proteins were cleaved by adding 400 µg PreScission protease per mL resin under agitation at 4°C overnight. Cleaved proteins were purified further by ion-exchange chromatography (NDP52: RESOURCE Q; LC3C: RESOURCE S, GE Life Sciences) using a linear salt gradient from 50 to 500 mM NaCl in pH 8.5 and 6.5 respectively, and by size exclusion chromatography (HiLoad 16/60 Superdex 75 column, GE Life Sciences) in 20 mM Tris, 200 mM NaCl, 5 mM DTT, pH 8.0 (NDP52) or pH 7.0 (LC3C).

NMR Analysis. NDP52 (21-141) was expressed as described above but with the following modification. Bacteria from a 40 mL overnight culture grown in LB medium were used to inoculate 4 L M9 minimum medium supplemented with 8 g of ^{15}N ammonium chloride, and 16 g of ^{13}C glucose for double-labeled samples, or 16g of glucose for single-labeled samples. Cells were lysed by sonication in NMR buffer (phosphate buffered saline (PBS), 18 mM Na_2HPO_4, 7 mM $NaH_2PO_4.xH_2O$, 150 mM NaCl, 5 mM DTT, pH 7.2). Purification was performed as described above, with the final gel filtration performed in NMR buffer. All NMR experiments were performed in PBS at neutral pH (pH 7.2) in the presence of 150 mM NaCl to mask non-specific interactions. All proteins were monodisperse in these buffer conditions at all analyzed protein concentrations.

NMR data were acquired using a 700MHz (^1H frequency) Bruker Avance2+ spectrometer with a cryogenic triple resonance probe, and a sample temperature of 298 K. All datasets were processed in TopSpin 2.1 (Bruker) and analyzed in Sparky (Goddard & Kneller, University of California, San Francisco). Standard triple resonance experiments (HNCACB, HNCA, CBCA(CO)NH, HNCACO and HNCO) were used to assign backbone resonances of ^{13}C,^{15}N-labeled NDP52, using a sample concentration of 150 µM. Unambiguous assignments were obtained for 109 of 116 non-Pro backbone correlations in the ^{15}N,^1H-HSQC, corresponding to 94% of the residues in the construct. All C-terminal residues comprising the CLIR peptide were unambiguously assigned. Exchange broadened resonances were identified from the intensity ratios at peak height maxima fitted in Sparky, without normalization.

Crystallization, data collection, phasing and refinement. Crystallization conditions were screened by the sitting-drop vapor diffusion method and optimized by hanging-drop vapor diffusion at 293 K. NDP52 (residues 21-141) crystals were obtained using 18.5 mg/mL protein, a reservoir solution containing 20% (w/v) polyethylene glycol (PEG) 4000, 5 mM cadmium chloride and 100 mM Tris (pH 8.5) after 5 days. The space group of these crystals was $P4_12_12$ and this crystal form was used for phasing. A similar crystallization condition lacking cadmium (24% (w/v)

polyethylene glycol (PEG) 4000, 100 mM Tris (pH 8.5)) resulted in crystals in a different space group ($P2_12_12_1$) that diffracted to high resolution (**Supplementary Table 1**).

NDP52 (21-141) in complex with LC3C (1-126) complex crystals were obtained using 10.0 mg/mL total protein in 1:1 molar ration, from a reservoir solution containing 16% (w/v) polyethylene glycol (PEG) 6000, 10 mM sodium citrate after 7 days. Prior to freezing in liquid nitrogen, crystals were briefly soaked in mother liquor containing additional 20% or 30% (v/v) glycerol (NDP52 or NDP52/LC3C complex, respectively).

For the purpose of phasing, a data set of an unliganded NDP52 SKICH crystal was collected to 2.7 Å resolution on a Rigaku FR-E+ SuperBright™ home source at 100 K. At the copper wavelength, the anomalous signal from ordered cadmium ions originating from the crystallization condition could be exploited for phasing. Further datasets were collected at European Synchrotron Radiation Facility (ESRF, Grenoble) beamline ID29. NDP52 SKICH crystals in a second space group diffracted to 1.35 Å resolution, and NDP52-LC3C complex crystals diffracted to 2.5 Å resolution (**Supplementary Table 1**).

Diffraction data was processed using iMosflm (Leslie, 2006) or XDS (Kabsch, 2010) and the CCP4 software suite (CCP4, 1994). The structure of NDP52 SKICH domain was determined by single anomalous dispersion methods using cadmium anomalous signal in autoSHARP (Bricogne et al., 2003). The structure of the NDP52-LC3C complex was solved by molecular replacement with Phaser (McCoy et al., 2005) using the NDP52 SKICH domain and LC3B (PDB-id 1ugm, (Sugawara et al., 2004)) as search models. Subsequent rounds of model building in Coot (Emsley and Cowtan, 2004) and refinement in Phenix (Adams et al., 2002) resulted in final statistics shown in **Supplementary Table 1**.

Fluorescence anisotropy. FITC-Ahx labeled NDP52 peptides (wild-type: ENEEDILVVTTQGE, V136S: ENEEDILVSTTQGE, I133W: ENEEDWLVVTTQGE) (Designer BioScience) were dissolved in Binding Buffer (50 mM Tris, 50 mM NaCl, pH 8.0). Purified ATG8 orthologues were serially diluted in Binding Buffer and mixed with equal volumes of 100 nM FITC-labeled NDP52 peptide. Fluorescent

measurements to detect change in light polarization of FITC-labeled NDP52 peptide were performed in 384-well format on a PheraStar plate reader with excitation and emission wavelengths at 488 nm and 518 nm, respectively. Anisotropy readings were normalized to a control of 10 µL of Binding Buffer mixed with 10 µL of 100 µM FITC-labeled NDP52 peptides. Binding data was analyzed with GraphPad Prism 5.

Supplemental References

Adams, P. D., Grosse-Kunstleve, R. W., Hung, L. W., Ioerger, T. R., McCoy, A. J., Moriarty, N. W., Read, R. J., Sacchettini, J. C., Sauter, N. K., and Terwilliger, T. C. (2002). PHENIX: building new software for automated crystallographic structure determination. Acta Crystallogr D Biol Crystallogr *58*, 1948–1954.

Barrios-Rodiles, M., Brown, K. R., Ozdamar, B., Bose, R., Liu, Z., Donovan, R. S., Shinjo, F., Liu, Y., Dembowy, J., Taylor, I. W., et al. (2005). High-throughput mapping of a dynamic signaling network in mammalian cells. Science *307*, 1621–1625.

Berrow, N. S., Alderton, D., Sainsbury, S., Nettleship, J., Assenberg, R., Rahman, N., Stuart, D. I., and Owens, R. J. (2007). A versatile ligation-independent cloning method suitable for high-throughput expression screening applications. Nucleic Acids Res *35*, e45.

Bloor, S., Maelfait, J., Krumbach, R., Beyaert, R., and Randow, F. (2010). Endoplasmic reticulum chaperone gp96 is essential for infection with vesicular stomatitis virus. Proc Natl Acad Sci USA *107*, 6970–6975.

Bloor, S., Ryzhakov, G., Wagner, S., Butler, P. J. G., Smith, D. L., Krumbach, R., Dikic, I., and Randow, F. (2008). Signal processing by its coil zipper domain activates IKK gamma. Proc Natl Acad Sci USA *105*, 1279–1284.

Bricogne, G., Vonrhein, C., Flensburg, C., Schiltz, M., and Paciorek, W. (2003). Generation, representation and flow of phase information in structure determination: recent developments in and around SHARP 2.0. Acta Crystallogr D Biol Crystallogr *59*, 2023–2030.

CCP4 (1994). The CCP4 suite: programs for protein crystallography. Acta Crystallogr D Biol Crystallogr *50*, 760–763.

Emsley, P., and Cowtan, K. (2004). Coot: model-building tools for molecular graphics. Acta Crystallogr D Biol Crystallogr *60*, 2126–2132.

Kabsch, W. (2010). Xds. Acta Crystallogr D Biol Crystallogr *66*, 125–132.

Leslie, A. G. (2006). The integration of macromolecular diffraction data. Acta Crystallogr D Biol Crystallogr *62*, 48–57.

McCoy, A. J., Grosse-Kunstleve, R. W., Storoni, L. C., and Read, R. J. (2005). Likelihood-enhanced fast translation functions. Acta Crystallogr D Biol Crystallogr *61*, 458–464.

Randow, F., and Sale, J. E. (2006). Retroviral transduction of DT40. Subcell Biochem *40*, 383–386.

Ryzhakov, G., and Randow, F. (2007). SINTBAD, a novel component of innate antiviral immunity, shares a TBK1-binding domain with NAP1 and TANK. EMBO J *26*, 3180–3190.

Thurston, T. L. M., Ryzhakov, G., Bloor, S., Muhlinen, Von, N., and Randow, F. (2009). The TBK1 adaptor and autophagy receptor NDP52 restricts the proliferation of ubiquitin-coated bacteria. Nat Immunol *10*, 1215–1221.
